# An efficient green protocol for the synthesis of 1,2,4,5-tetrasubstituted imidazoles in the presence of ZSM-11 zeolite as a reusable catalyst†

**DOI:** 10.1039/d1ra07984k

**Published:** 2022-02-02

**Authors:** Sudarshan S. Dipake, Vijayanand D. Ingale, Sonali A. Korde, Machhindra K. Lande, Anjali S. Rajbhoj, Suresh T. Gaikwad

**Affiliations:** Department of Chemistry, Dr Babasaheb Ambedkar Marathwada University Aurangabad 431004 India gaikwadsuresh12@gmail.com

## Abstract

In this study, we have synthesized a series of ZSM-11 zeolite catalysts using tetrapropyl ammonium hydroxide as a structure-directing agent through a highly efficient hydrothermal method. The series of catalysts were studied by different techniques such as FT-IR spectroscopy, XRD, FE-SEM, HR-TEM, EDS, pyridine-FT-IR spectroscopy, and BET analysis. We focused on varying reaction time intervals from 18 to 48 hours to investigate the effect on catalytic activities of the synthesized series of catalysts. The percentages of aluminum increased in the framework of zeolites with increasing crystallinity, surface area, external surface area, and acidity in the series of ZSM-11 zeolites by increasing the time from 18 to 48 h. Then, we studied the catalytic activity of a series of ZSM-11 zeolites and found that the ZSM-11 zeolite (48 h) possesses higher catalytic activity towards the synthesis of 1,2,4,5-tetrasubstituted imidazoles under solvent-free conditions. The present protocol scored well with excellent yield, short reaction time, clean reaction profiles, low catalyst loading, and no tedious workup. The catalyst (ZSM-11 zeolite 48 h) was recycled and reused in five runs without any considerable loss of activity and product yield.

## Abstract 2

We synthesized a series of ZSM-11 zeolite catalysts, investigating the effect of varying the reaction time interval from 18 to 48 h. The ZSM-11 zeolite (48 h) has higher catalytic activity for the synthesis of 1,2,4,5-tetrasubstituted imidazoles.

## Graphical abstract

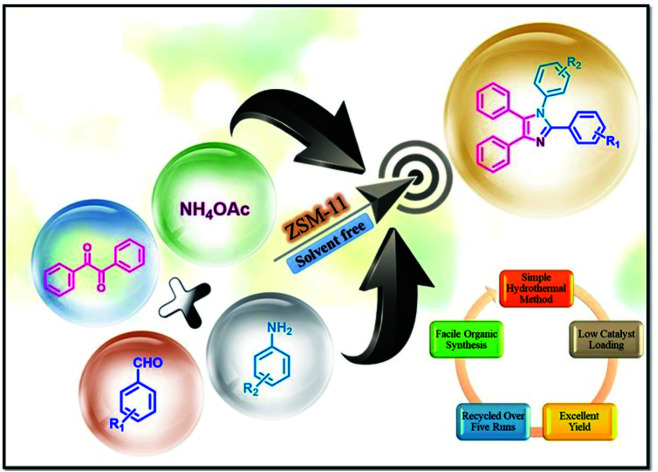

## Introduction

Multicomponent reactions (MCRs) have emerged as powerful and efficient bond-forming tools because they have attracted considerable attention in modern organic and medicinal chemistry. MCRs are highly recommended for the synthesis of products without any intermediates by minimizing the tedious workup and reaction time. The improvement of known MCRs is currently under intense focus because of their vital scope of application in modern drug design.^1–4^ Substituted imidazoles are a class of nitrogen-containing five-member ring system heterocycles, in which nitrogen atoms are one of the most imperative motifs established in a large number of pharmacologically active compounds and natural products.^5^ Imidazoles are important bioactive heterocyclic core fragments that have proved to possess a spectrum of biological activities, covering antitumor, anti-allergic, anti-tubercular, antifungal, antibacterial, anti-biotic, anti-cancer, anti-HIV, and antiviral activities.^6^ Many multi-substituted imidazoles act as p38 MAP kinase inhibitors, glucagon receptors, anti-inflammatory agents, therapeutic agents, and plant growth regulators.^1,7–9^ The potency and broad applicability of the imidazole pharmacophore might be attributed to its hydrogen bond donor–acceptor capability along with its high affinity for metals, which exist in many protein active sites.^10^ Furthermore, they are essential fragments of several biological and drug molecules such as histidine, histamine, biotin, losartan, olmesartan, eprosartan, and trifenagrel.^11^

There are several synthetic protocols for the preparation of imidazoles *via* MCRs of 1,2-diketone, aldehyde, aniline, and ammonium acetate with alteration of catalysts and parameters including molecular iodine,^12^l-proline,^10^ DABCO,^13^ H_2_SO_4_,^14^ CH_3_COOH,^15^ K_5_CoW_12_O_40_·3H_2_O,^16^ urea/hydrogen peroxide,^17^ graphene oxide–chitosan bio nanocomposite,^18^ heteropoly acid,^19^ HClO_4_–SiO_2_,^20^ and diethyl bromophosphate.^21^ Nevertheless, several of these synthetic protocols are not eco-friendly and suffer from one or more drawbacks such as applying hazardous volatile organic solvents, use of toxic reagents, low yield, by-products, prolonged reaction time, tedious workup methods, high cost and non-recoverability of the catalyst. Therefore, overcoming these drawbacks and developing eco-friendly protocols are still highly desirable.

**Scheme 1:**
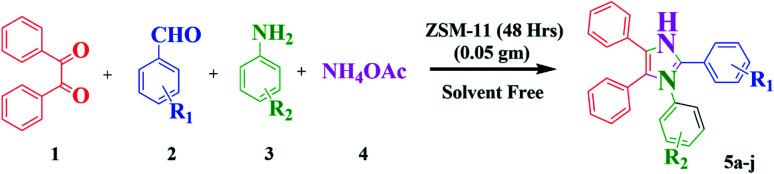
Synthesis of 1,2,4,5-tetrasubstituted imidazole derivatives.

Nowadays, the development of eco-friendly protocol for synthesizing imidazoles has attracted continuous interest in the medicinal field. Considering these aspects and the continuations of our research work using the new zeolite catalyst for the development in synthetic methodology,^6,7^ here, we report the preparation, characterization, and catalytic application of ZSM-11 zeolite as an efficient and reusable heterogeneous catalyst in MCR of 1,2,4,5 tetrasubstituted imidazole derivatives using 1,2-diketone, aldehyde, aniline, and ammonium acetate under solvent-free conditions (Scheme 1). ZSM‐11 zeolite has two crossing straight channels (0.53 nm × 0.54 nm, 0.53 nm × 0.54 nm) and is topologically analogous to ZSM-5, which is receiving considerable attention because of its excellent performances as solid acid and shape‐selective catalysts in various industrial processes. The use of ZSM-11 is expected to give an efficient catalytic activity along with its moderate acidity as a heterogeneous catalyst.^22^ The use of heterogeneous catalysts in various organic transformations is of great interest because they have many advantages such as suitable acidity, thermal stability, insolubility, simple work-up, recyclability, and being environmentally safe.^23–28^

In this work, we studied the influence of time intervals on the morphology and catalytic activity of ZSM-11 and we found interesting results. The percentage of aluminum increased in the framework of zeolites with increasing crystallinity, surface area, external surface area, and acidity in the series upon increasing the time from 18 to 48 h. Here, we noted that the ZSM-11 zeolite (48 h) possesses a higher catalytic activity towards the synthesis of 1,2,4,5-tetrasubstituted imidazole under solvent-free conditions. The present protocol scored well in excellent yield, short reaction time, clean reaction profiles, low catalyst loading, and no tedious work-up. The catalyst (ZSM-11 zeolite, 48 h) was recycled and reused in five runs without any considerable loss of activity and product yield (Scheme 1).

## Experimental details

### Materials and methods

All reagents were purchased from Sigma Aldrich and used without further purification. Synthesized materials were characterized using different techniques such as Fourier transform infrared (FT-IR) spectroscopy using a Shimadzu FT-IR 8300 spectrophotometer for functional integrity. Powder X-ray diffraction (XRD) was performed on a Bruker D8-Advanced diffractometer for studying crystallinity. FE-SEM imaging was performed on a Nova Nano SEM NPEP303 instrument for elemental mapping and energy dispersive X-ray spectroscopy (EDS) was performed on Objects 8724 for the elemental content. The specific surface area and pore volume were calculated using a Quantachrome instrument version 3.01 and by Brunauer–Emmett–Teller (BET) analysis. The morphology of the samples was studied using a scanning electron microscope (SEM), Nova Nano SEM NPEP303, and high-resolution transmission electron microscopy (HR-TEM) on a JEOL JEM 2100 Plus microscope. The reaction progress was monitored using thin-layer chromatography (TLC). Melting points of the substrates were determined using the assembly of the open capillary tube and are uncorrected. The ^1^H NMR (400 MHz) and ^13^C NMR (101 MHz) were run on a Bruker (400 MHz) spectrometer using tetramethylsilane (TMS) as an internal reference.

### Catalyst preparation

**Scheme 2:**
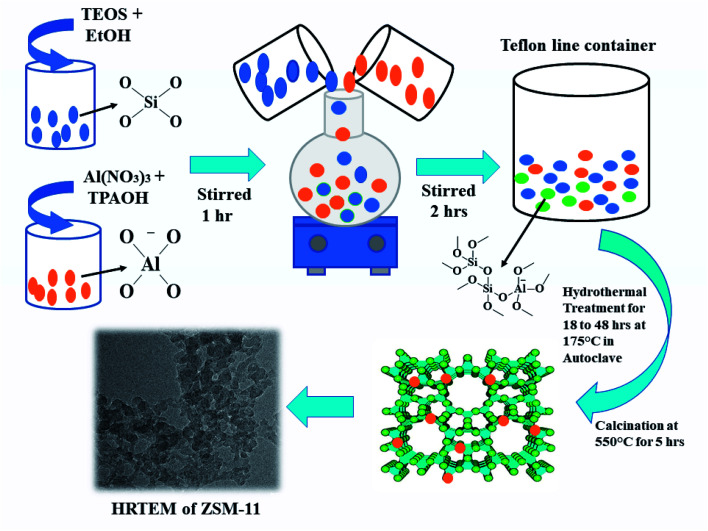
Schematic representation of the synthesis of the ZSM-11 zeolite.

ZSM-11 catalyst was prepared *via* hydrothermal pathway as shown in Scheme 2. At first, 23 ml of tetraethyl orthosilicate (TEOS) silica source in 20 ml of deionized water (DI) and 20 ml of alcohol was stirred vigorously for one hour to form the silicate solution. In another beaker 1.73 g of aluminum nitrate (Al(NO_3_)_3_) in 20 ml of deionized water was taken. Then, 27 ml of tetra propyl ammonium hydroxide (TPAOH) base as a structure-directing agent was added dropwise to aluminum nitrate solution and stirred vigorously for one hour at room temperature up to pH 12 to form alumina solution. Finally, the alumina solution was mixed dropwise into the silicate solution with constant vigorous stirring for two hours to obtain a transparent viscous gel. The resulting reaction mixture was transferred in an autoclave for hydrothermal treatment at 175 °C for 18 h. The solid product was separated by filtration and frequently washed with deionized water followed by drying in an oven at 120 °C for 2 h and, subsequently, the product was calcined at 550 °C for 5 h in a muffle furnace to remove the template. We applied the same procedure for preparing different batches of ZSM-11 but at different times such as 24, 36, and 48 h. For comparison, all the synthesized ZSM-11 were characterized using different analysis techniques and then utilized in the synthesis of 1,2,4,5-tetrasubstituted imidazole derivatives.

### General procedure for the synthesis of 1,2,4,5-tetrasubstitutedimidazole derivatives

All the ZSM-11 zeolites were activated by heating at 550 °C for 5 h before placing them into the reaction mixture. In a typical reaction, the suspension of benzil (1 mmol), aldehyde (1 mmol), aniline (1 mmol), and ammonium acetate (3 mmol) were added in 50 ml round bottom flask and were heated in an oil bath at 110 °C with continuous stirring for 30 minutes under solvent-free conditions in the presence of ZSM-11 zeolite (0.05 g) as a catalyst. The confirmation of completion of the reaction was monitored using TLC using petroleum ether : ethyl acetate (6 : 4) as a solvent system. After the completion of the reaction, the reaction mass was cooled at room temperature, followed by ethanol was added to the crude product. The spent catalyst was separated from the residual reaction mixture by filtration and then washed with acetone. The obtained crude product was recovered by solvent evaporation and further purified by recrystallizing in ethanol to obtain the pure product. We applied the same procedure for the synthesis of other derivatives of 1,2,4,5-tetrasubstituted imidazoles (5a–j). The desired products were confirmed by comparing their physical and spectral data with those of authentic samples, the data are shown in Table 4.

## Results and discussion

**Fig. 1:**
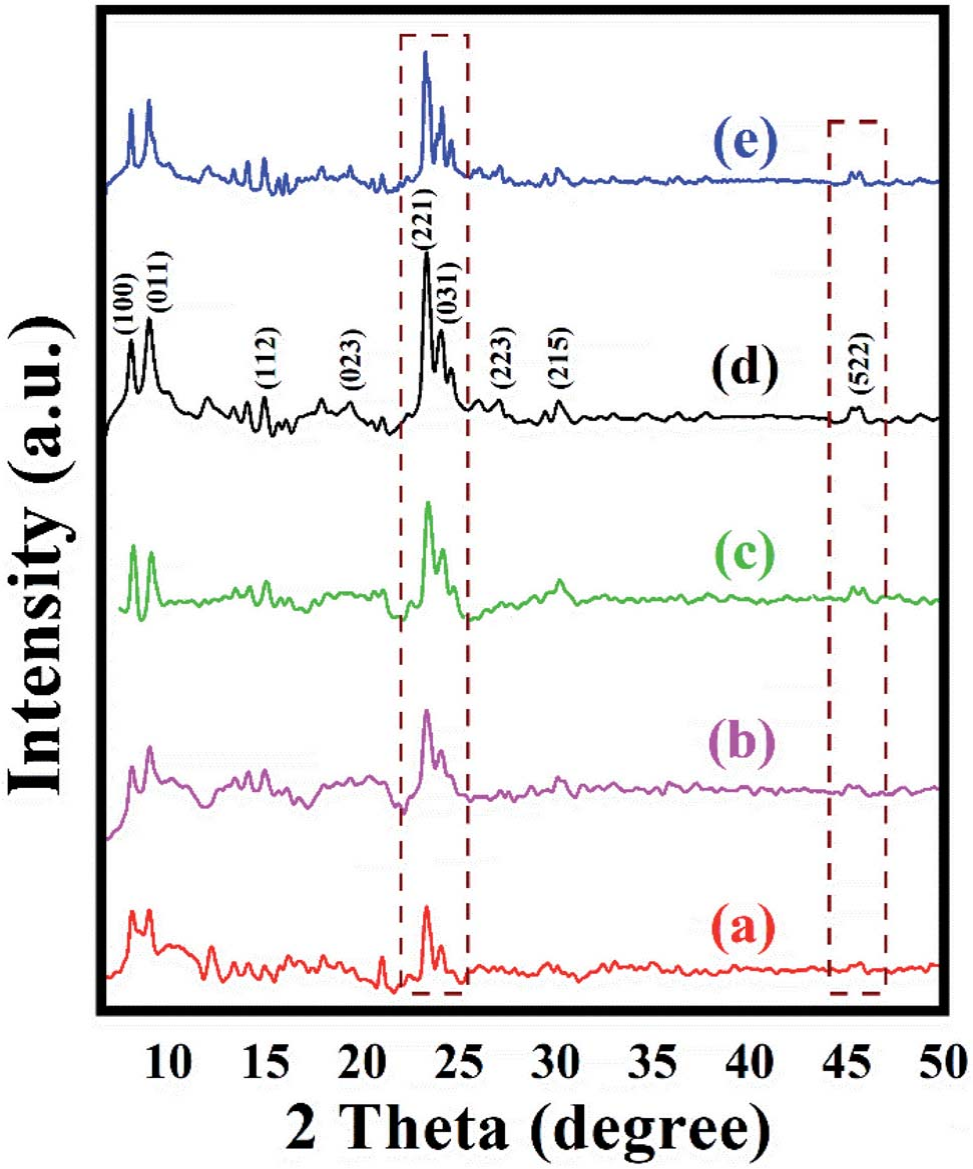
XRD pattern of the synthesized ZSM-11 zeolites at various time intervals (a) 18 h, (b) 24 h, (c) 36 h, (d) 48 h and (e) recycled ZSM-11 (48 h).

XRD data were collected to study the crystalline phase as well as the ordered structure of the prepared zeolites. XRD patterns of the series of ZSM-11 zeolites prepared at different time intervals are shown in Fig. 1. The intensity of the diffraction peaks increased regularly with increasing time during the hydrothermal reaction. The less intense diffraction peaks of ZSM-11 zeolite were observed in 18 h. Then, diffraction peaks were gradually increased with the increasing time from 18 to 48 h. Afterward, increasing the time did not affect the intensity of diffraction peaks indicating that the formation of the crystals was complete in 48 h. As the crystallization time increases, silicate units may interact with the remaining surfactant molecules/aluminum and the formation of new surfactant/silica/alumina aggregates, resulting in the construction of larger and improved long-range ordering crystallites. The XRD and EDS results revealed that the crystallinity and insertion of aluminum in a framework of ZSM-11 zeolites are dependent on the time of the hydrothermal reaction. High intensity and low background diffraction peaks indicated that the zeolite ZSM-11 (48 h) was in a well-crystallized form. The diffraction peaks for the series of zeolite samples exhibited at 2*θ*° 8.06, 9.01, 14.95, 23.30, 24.01 and 45.4 with a corresponding plane (100), (011), (112), (221), (031) and (522), respectively, which confirmed near to the ZSM-11 phase structure with MEL-type topology. Generally, the pure phase of the ZSM-11 is difficult to prepare in the absence of ZSM-5 intergrowth on ZSM-11 particles. It was previously reported that the appearance of doublet peaks in between the range 23–25*θ*° and a single reflection peak at 2*θ*° of 45.40 confirmed the absence of ZSM-5 phase in ZSM-11 particles.^29–31^ The international zeolite association data also confirmed that the highest intense peak at 23.30*θ*° is near about the ZSM-11 phase and MEL topology.^32^

**Fig. 2:**
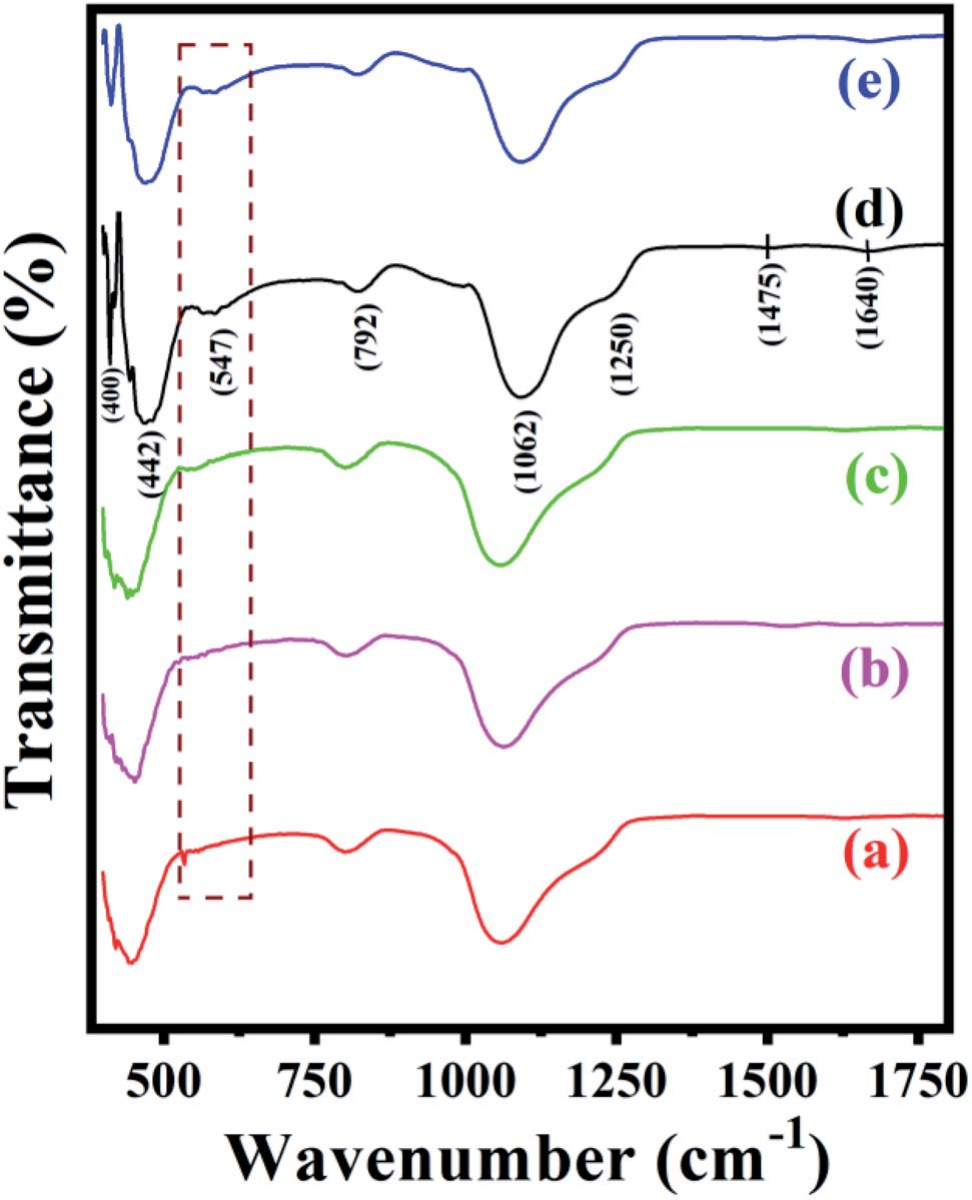
FT-IR spectra of the synthesized ZSM-11 zeolites at various time intervals (a) 18 h, (b) 24 h, (c) 36 h, (d) 48 h and (e) recycled ZSM-11 (48 h).

The FT-IR spectra of synthesized ZSM-11 zeolites at different time intervals such as 18, 24, 36, and 48 h are shown in Fig. 2. The series of zeolite samples exhibited characteristic absorption bands at 400, 442, 547, 792, 1062, 1250, 1475, and 1640 cm^−1^. The absorption bands at 792, 1062, and 1250 cm^−1^ are attributed to the external symmetric, internal asymmetric, and external asymmetric stretching of the Si–O–T linkage in the framework, respectively. The bending vibration of TO_4_ (T = Si or Al) in the framework was recorded at around 442 cm^−1^, while the vibration band at around 547 cm^−1^ and 1250 cm^−1^ indicated the presence of double five rings (D5R) of the characteristic structure of pentasil family zeolite. Interestingly, ZSM-11 type zeolite shows the intense band at near 550 cm^−1^ whose position changes with the composition of aluminum. Nevertheless, the absence of the absorption band around 550 cm^−1^ indicates that the ZSM-11 zeolite type framework is not present in the samples.^31,33–36^ The overall absorption bands for the series of zeolite samples were similar to one another. With increased reactions time from 18 to 48 h, we observed that the intensity of the peak at 547 cm^−1^ was increased due to the percentage of aluminum insertion increases in the zeolite framework.^37^ Hence, based on observed FT-IR and EDS results, it is concluded in this work that the synthesis of ZSM-11 zeolite type structure with MEL topology needs 3.17% of aluminum as well as 48 h for the completion of the reaction.

**Fig. 3:**
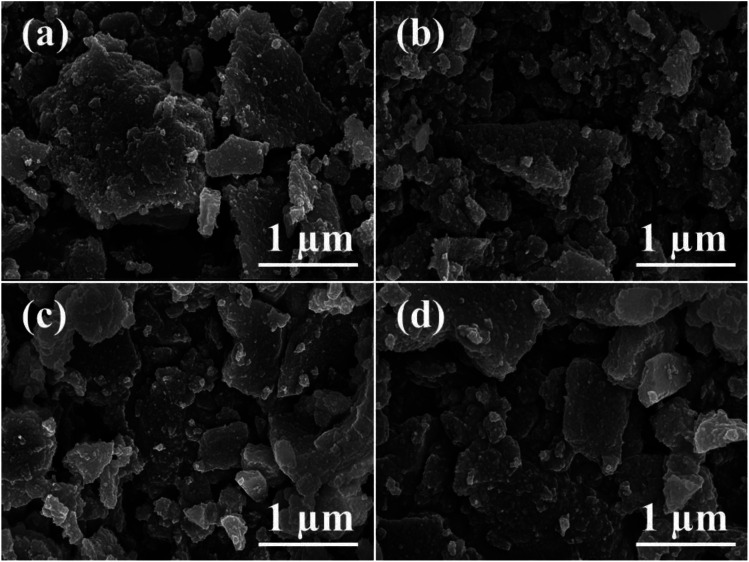
FE-SEM images of the synthesized ZSM-11 zeolites at various time intervals (a) 18 h, (b) 24 h, (c) 36 h, and (d) 48 h.

The FE-SEM images of a series of ZSM-11 zeolites are shown in Fig. 3. ZSM-11 zeolites have typical sheet-like morphology with a smooth surface, crystalline rough edges, and compact surface. The exclusive presence of inherent micropores and mesopores in the samples was demonstrated. The primary crystals were aligned in a parallel fashion nearer to the crystals giving a special morphology of polycrystalline aggregates of ZSM-11 zeolites.

**Fig. 4:**
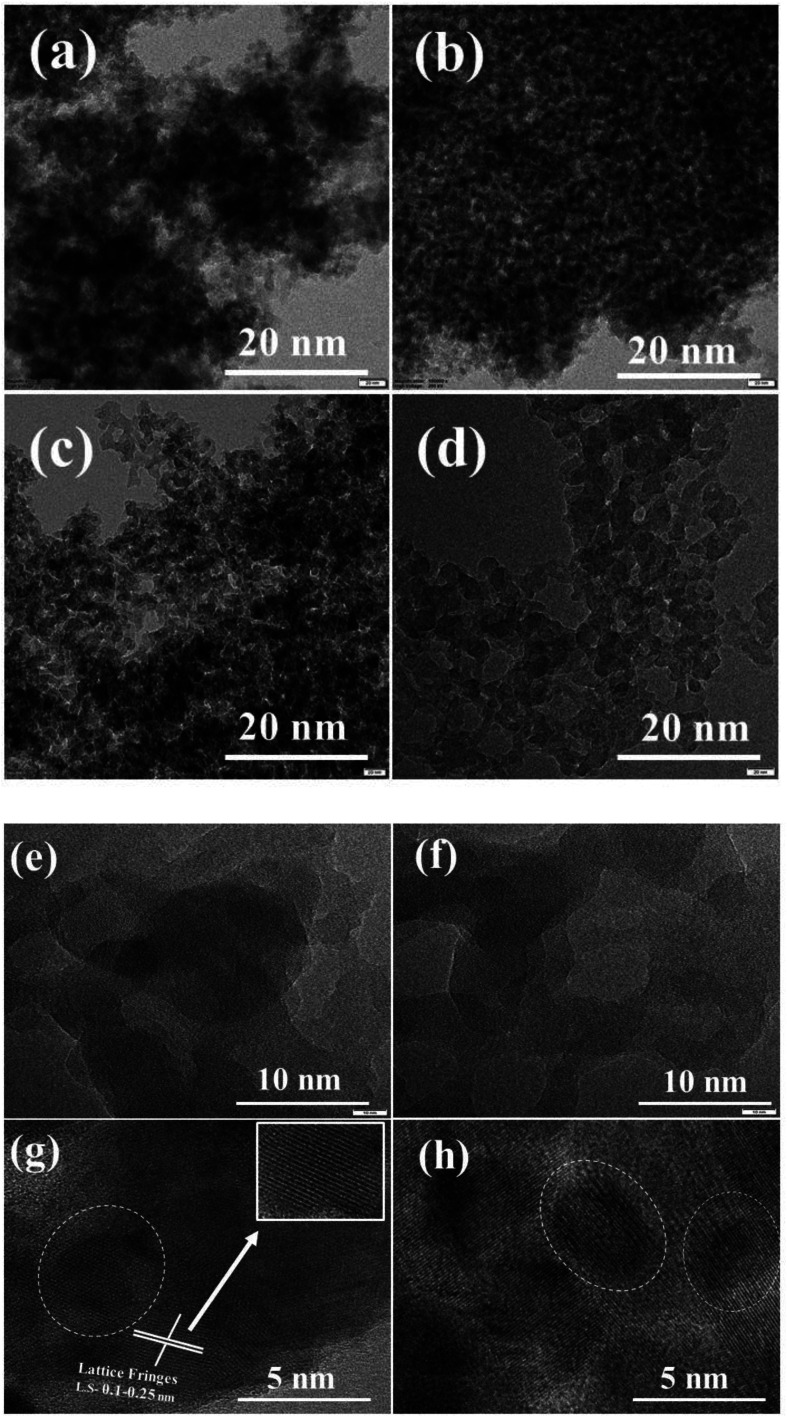
HR-TEM images of the synthesized ZSM-11 zeolites at various time intervals, (a) 18 h, (b) 24 h, (c) 36 h, (d) 48 h and (e–h) HR-TEM images of the ZSM-11 zeolite 48 h.

HR-TEM images of the series of ZSM-11 zeolites with different times are presented in Fig. 4. The intergrowth of crystal in the framework is well crystalline, which is revealed through the intense diffraction peaks of the XRD pattern shown in Fig. 1. The regular orientations of lattice fringes in the framework reveal the presence of micropores. It was noted that some irregular orientation of lattice fringes was also observed due to the formation of mesopores in the framework.^38^Fig. 4g shows that the lattice fringes were also implanted in the mesoporous framework (white circle of a dotted line). The interspace between the crystalline particles could be visualized may be due to the formation of a mesoporous structure (Fig. 4e and f).^39–42^ Based on the obtained results of BET analysis, we observed an increased external surface area regularly with increasing time from 18 to 48 h^43^ (Table 1). Hence, ZSM-11 zeolite (48 h) shows a high external surface area as compared to the rest of the zeolite samples (18, 24, 36 h). Fig. 4g and h indicates that the zeolite samples possess lattice fringes of micropores, and the lattice spacing was approximately between 0.1 to 0.25 nm. The intracrystalline microporous lattice fringes cannot originate perfectly in many zeolite frameworks and this common phenomenon exists in many hierarchical zeolites.^44^ The HR-TEM and FE-SEM images are consistent with XRD, FT-IR, and BET results.

**Table 1:** The textural parameters of the prepared series of the ZSM-11 zeolite samples with different times (18, 24, 36, 48 h)

ZSM-11 (h)	*S*_BET_ a (m^2^ g^−1^)	*S*_ext_ b (m^2^ g^−1^)	*S*_micro_ c (m^2^ g^−1^)	*D*_average_ d (nm)	*V*_total_ e (cm^−1^ g^−1^)
18	319.363	306.311	13.052	9.65	0.770
24	330.188	328.191	1.997	10.08	0.893
36	339.950	335.595	4.355	10.05	0.892
48	369.791	349.937	19.854	8.02	0.762

a BET surface area.

b External surface area.

c Micropore area.

d Average pore diameter, and

e Total pore volume.

The textural properties of a series of ZSM-11 zeolites at different times (18, 24, 36, 48 h) were analyzed using nitrogen adsorption–desorption. The given data show the surface area (*S*_BET_), external surface (*S*_ext_), micropore area (*S*_micro_), and total pore volume (*V*_total_) of ZSM-11 zeolite samples as summarised in Table 1. The zeolite samples showed a gradual increase in surface area (*S*_BET_), and external surface area (*S*_ext_) by increasing the time from 18 h to 48 h. It can be observed that the ZSM-11 zeolite (48 h) possesses the highest surface area (369.791 m^2^ g^−1^) as well as external surface area (349.937 m^2^ g^−1^) than the rest of the zeolite samples, which may be important for the catalytic performance, while ZSM-11 zeolite (18 h) has the lowest surface area and external surface area (319.363 and 306.311 m^2^ g^−1^), and the rest of ZSM-11 zeolite samples prepared at 24 and 36 h have intermediate surface areas (330.188 and 339.950 m^2^ g^−1^), and external surface areas (328.191 and 335.595 m^2^ g^−1^), respectively. The increased available surface area and external surface area can be recognized as an additional feature contributing to the better catalytic performance towards the synthesis of 1,2,4,5-tetrasubstituted imidazole derivatives.

**Fig. 5:**
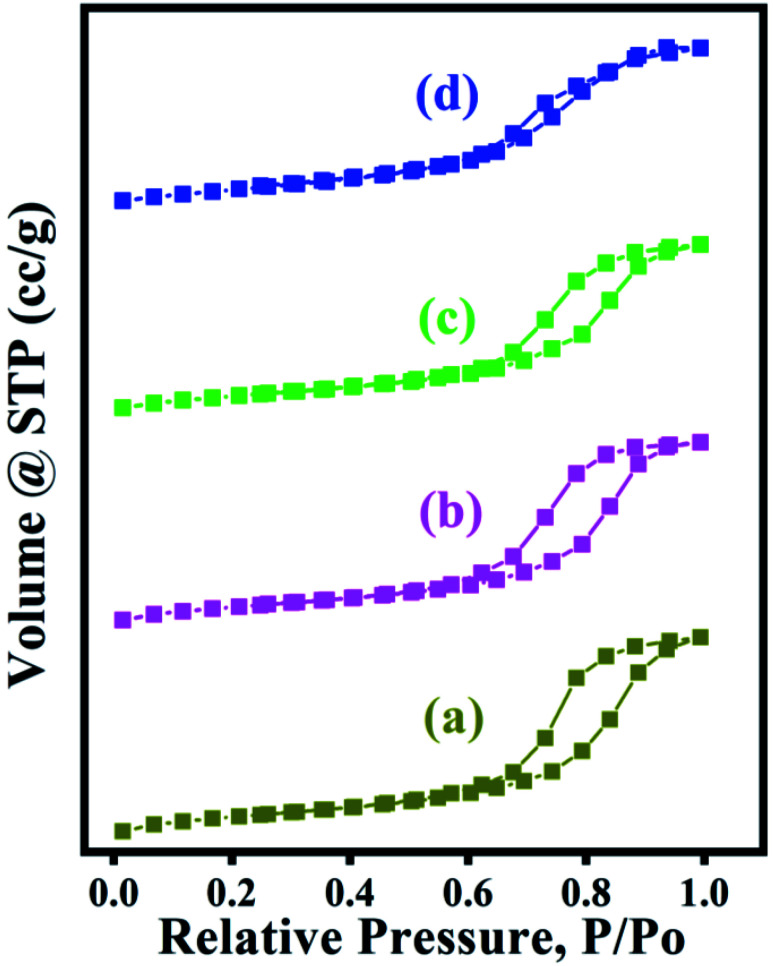
N_2_ adsorption–desorption isotherms of a series of ZSM-11 zeolites at various (18–48 h) times (a) 18 h, (b) 24 h, (c) 36 h, (d) 48 h.

N_2_ adsorption and desorption isotherms of a series of zeolites (Fig. 5) showed the type IV isotherms curves having an H1 hysteresis loop. The steep increase at very low relative pressure *P*/*P*_0_ < 0.02 and the hysteresis loop at relative pressure between *P*/*P*_0_ of 0.6–1.0 indicate the presence of both micropores and mesopores in a series of zeolites.

**Fig. 6:**
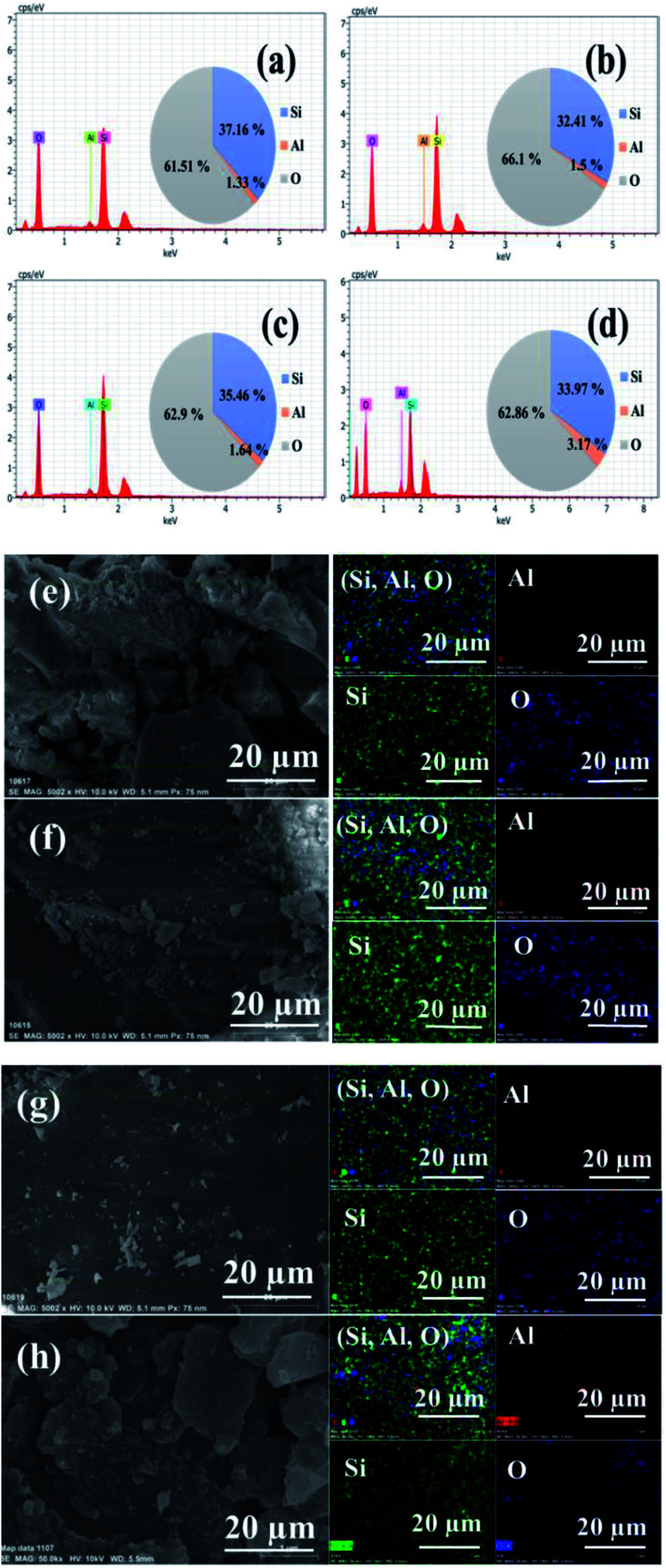
EDS of the series of ZSM-11 zeolites: 18 h (a), 24 h (b), 36 h (c), and 48 h (d). Elemental mapping of ZSM-11 zeolites: 18 h (e), 24 h (f), 36 h (g), and 48 h (h).

The composition of ZSM-11 zeolites was analyzed using an energy dispersive spectrometer (EDS), which unambiguously demonstrates the co-existence of aluminum, silicon, and oxygen elements in zeolite samples. The atomic mass percentage of aluminum was regularly increased from 1.33 to 3.17% in zeolite samples by increasing the time of reaction from 18 to 48 h. The elemental mapping also confirmed that the composition of Si, Al, O was uniformly distributed in the framework of the ZSM-11 zeolites, as shown in Fig. 6.

**Fig. 7:**
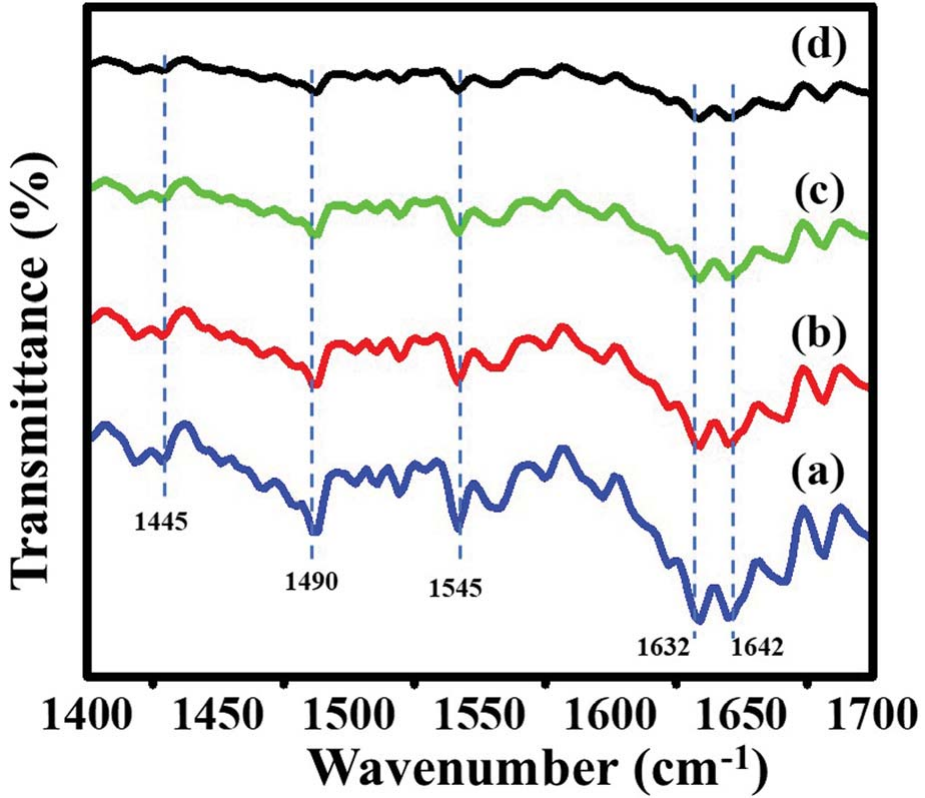
Pyridine IR spectroscopy of the ZSM-11 zeolites: (a) 48 h, (b) 36 h, (c) 24 h, and (d) 18 h.

The nature of acid sites on calcined zeolites was characterized by using pyridine-IR spectroscopy. This technique provides useful information about Brønsted and Lewis acidic sites. IR spectra of adsorbed pyridine on ZSM-11 zeolites are shown in Fig. 7, in which, two bands appeared at 1445 and 1632 cm^−1^ related to Lewis-bonded pyridine (*i.e.*, Lewis acidic sites), two bands at 1545 and 1642 cm^−1^ assigned to pyridinium cations (*i.e.*, Brønsted acid sites) and the band at 1490 cm^−1^ assigned to the synergetic result on pyridine adsorbed on both Lewis and Brønsted.^29,45–48^ Pyridine-IR spectra of a series of ZSM-11 zeolites show that the peaks intensity of Lewis acid sites and Brønsted acid sites increases with time from 18 h to 48 h. Hence, we conclude that the percentage insertion of aluminum was increased in the framework of zeolites by increasing time from 18 to 48 h, due to which the acidity of zeolites increased simultaneously.^46,49^

**Fig. 8:**
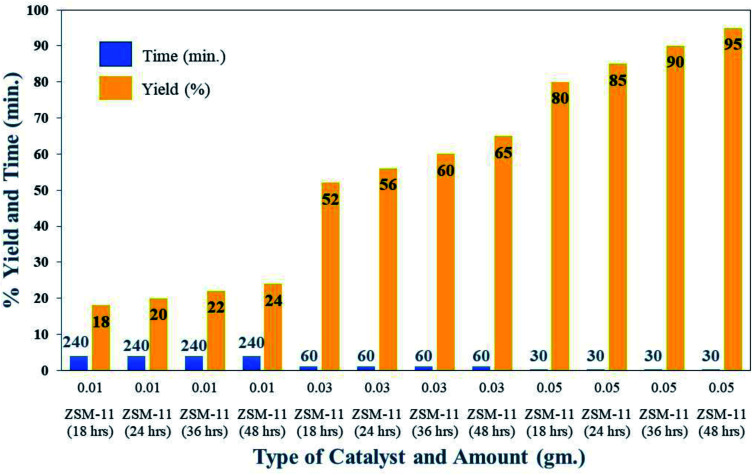
Optimisation of catalyst amounts for the synthesis of 1,2,4,5-tetrasubstituted imidazole derivatives (5f) under solvent-free conditions at 110 °C.

TGA and DTA curves of the synthesized ZSM-11 zeolites using TPAOH as a structure-directing agent are illustrated in the ESI† shown in Fig. 8. In the thermogram, the total weight loss of synthesized ZSM-11 zeolites is 18.50%. When the sample is heated from room temperature to 800 °C, the weight loss below 200 °C is due to the presence of adsorbed water or guest molecule, and the loss from 200 to 700 °C is likely due to the decomposition of the template present in zeolite channels.

Based on the above-obtained data on XRD, FT-IR, BET, EDS, and pyridine-IR spectra, we conclude that the more insertion of aluminum in the framework of zeolites with increasing time results in an increase in crystallinity and surface area, external surface area, and Lewis as well as Brønsted acid sites.

### Optimization of catalyst

To obtain the optimum parameters for the synthesis of 1,2,4,5-tetrasubstituted imidazoles in the presence of ZSM-11 zeolites, we monitored the model reaction under various conditions such as the catalyst amount, temperature, and solvent. Several solvents were studied to examine the activity of the synthesized zeolites in the ZSM-11 series and found that the solvent-free condition was the optimum condition and ZSM-11 zeolite (48 h) was the most active catalyst in the series (Table 2). Solvent-free conditions were the best because the availability of reactants with active sites of the catalyst may be higher in the reaction mixture. Fig. 8 shows the optimized amount of the ZSM-11 zeolite in the series concerning the reaction time and yield percentage in solvent-free conditions. The results indicated that 0.05 g of ZSM-11 zeolite (48 h) gives the highest yield in 30 minutes (Fig. 8). Table 2 also includes the optimized reaction temperature concerning the reaction time, yield percentage, and type of catalyst. The results show that 110 °C is the optimum temperature in the presence of ZSM-11 zeolite (48 h) catalyst under solvent-free conditions (Table 2).

**Table 2:** Optimization studies of the current series of catalysts with different reaction conditions for the synthesis of 1,2,4,5-tetrasubstituted imidazole derivatives (5f)^a^

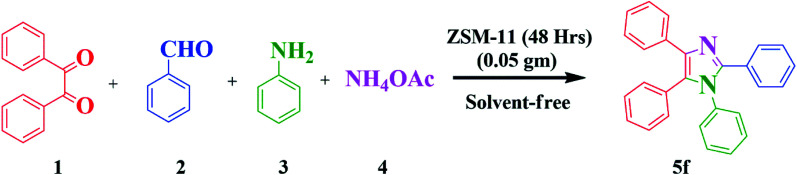						
Entry	Zeolite catalyst	Amount (g)	Solvent	Condition (temp.)	Time (min)	Yield^b^ (%)
1	ZSM-11 (48 h)	0.05	Water	Reflux	120	20
2	ZSM-11 (48 h)	0.05	Ethanol	Reflux	120	40
3	ZSM-11 (48 h)	0.05	Chloroform	Reflux	120	45
4	ZSM-11 (48 h)	0.05	DMF	Reflux	120	50
5	ZSM-11 (18 h)	0.05	Solvent-free	RT	240	Trace
6	ZSM-11 (24 h)	0.05	Solvent-free	RT	240	Trace
7	ZSM-11 (36 h)	0.05	Solvent-free	RT	240	Trace
8	ZSM-11 (48 h)	0.05	Solvent-free	RT	240	Trace
9	ZSM-11 (18 h)	0.05	Solvent-free	80 °C	60	60
10	ZSM-11 (24 h)	0.05	Solvent-free	80 °C	60	65
11	ZSM-11 (36 h)	0.05	Solvent-free	80 °C	60	70
12	ZSM-11 (48 h)	0.05	Solvent-free	80 °C	60	75
13	ZSM-11 (18 h)	0.05	Solvent-free	100 °C	40	70
14	ZSM-11 (24 h)	0.05	Solvent-free	100 °C	40	75
15	ZSM-11 (36 h)	0.05	Solvent-free	100 °C	40	80
16	ZSM-11 (48 h)	0.05	Solvent-free	100 °C	40	85
17	ZSM-11 (48 h)	0.05	Solvent-free	110 °C	30	95
18	ZSM-11 (48 h)	0.05	Solvent-free	120 °C	30	95
19	ZSM-11 (48 h)	—	Solvent-free	110 °C	240	—

a Reaction conditions: benzil (1.0 mmol), benzaldehyde (1.0 mmol), aniline (1.0 mmol), NH_4_OAc (3.0 mmol), ZSM-11 zeolite catalyst of time (18, 24, 36, and 48 h) (0.05 g).

b Isolated yield.

Based on the observed characterization results on the synthesized ZSM-11 zeolite from the series, we conclude that the surface area and acidity of catalysts increased by increasing time from 18 to 48 h. The catalyst ZSM-11 zeolite at 48 h with a high surface area and high acidity showed higher activity in the series of catalysts under solvent-free conditions. Therefore, we chose ZSM-11 zeolite (at 48 h) as the best catalyst and 0.05 g as the optimized amount under solvent-free conditions at 110 °C temperature.

Table 3 shows the comparative study of current protocols with the reported protocols for the synthesis of 1,2,4,5-tetrasubstituted imidazole derivatives. Table 3 shows the efficiency and merits of several reported catalysts. Despite their tremendous success, several of these suffer from one or more thoughtful drawbacks, such as long reaction times, lower yield, use of toxic catalysts, complex work-up procedures, and high catalyst loading. Therefore, this approach is clean and environmentally friendly for synthesizing 1,2,4,5-tetrasubstituted imidazole derivatives with purity and higher yield in a short time.

**Table 3:** Comparison of the current catalyst with reported catalysts for the synthesis of 1,2,4,5-tetrasubstituted imidazole derivatives^a^

Entry	Catalyst	Solvent	Temp. (°C)	Time (min)	Yield^b^ (%)	References
1	BF_3_·SiO_2_	Solvent-free	140	120	92	50
2	SiO_2_·NaHSO_4_	Solvent-free	140	120	92	51
3	TiCl_4_·SiO_2_	Solvent-free	110	190	75	52
4	PEG-400	Solvent-free	110	360	86	53
5	Glycerol	Solvent-free	90	180	96	54
6	l-Proline in	MeOH	60	540	78	10
7	Fe_3_O_4_–PEG–Cu	Solvent-free	110	55	96	55
8	InCl_3_·3H_2_O	MeOH	25	498	82	56
9	DABCO	*t*-BuOH	60	800	92	13
10	K_5_CoW_12_O_40_·3H_2_O	Solvent free	140	160	95	16
11	[(CH_2_)_4_SO_3_HMIM][HSO_4_]	Solvent free	140	120	94	57
12	ZSM-11 zeolite	Solvent-free	110	30	95	Present

a Reaction conditions: benzil (1.0 mmol), aldehyde (1.0 mmol), aniline (1.0 mmol), NH_4_OAc (3.0 mmol), ZSM-11 zeolite catalyst of time 48 h (0.05 g).

b Isolated yield.

Moreover, the impact of the catalyst on a variety of aldehydes and amines for synthesizing 1,2,4,5-tetrasubstituted imidazole derivatives was assessed. The outcomes summarized in Table 4 reveal that the aromatic aldehydes and amines containing electron-donating groups and electron-withdrawing groups on their aromatic ring afforded the associated desired products in high to excellent yield. The product yield of different aromatic aldehydes with electron-withdrawing substituents was higher than those with electron-releasing substituents, and the product yield of the aromatic amines with electron-releasing groups was higher than those with electron-withdrawing groups. Finally, it is noteworthy that the best results were observed at 110 °C with a ratio of diverse aldehyde (1 mmol) : benzil (1 mmol) : different aniline (1 mmol) : ammonium acetate (3 mmol) : ZSM-11 zeolite (48 h) (0.05 g) equal to 1 : 1 : 1 : 3 : 0.05 under solvent-free conditions.

**Table 4:** Synthesis of 1,2,4,5-tetrasubstituted imidazole derivatives using the ZSM-11 zeolite (48 h) catalyst under solvent-free conditions^a^

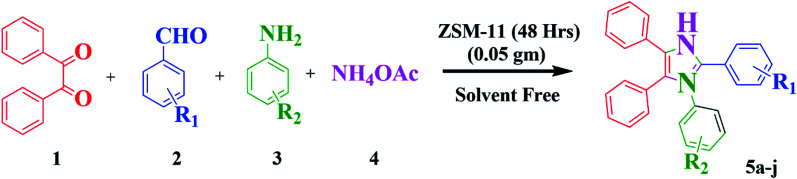						
Entry	R_1_	R_2_	Product	Yield^b^ (%)	M.p. (obsd) (°C)	M.p. (lit.) (°C)
1	4-CH_3_	C_6_H_5_	5a	92	184–186	185–187 ref. 58
2	2-OMe	C_6_H_5_	5b	94	208–210	207–211 ref. 59
3	C_6_H_5_	4-CH_3_	5c	95	284–285	285–289 ref. 6
4	4-OH	C_6_H_5_	5d	92	280–282	282–284 ref. 11
5	4-Cl	C_6_H_5_	5e	93	160–161	160–163 ref. 60
6	C_6_H_5_	C_6_H_5_	5f	95	215–217	216–218 ref. 61
7	4-OCH_3_	C_6_H_5_	5g	92	182–184	183–185 ref. 58
8	4-NO_2_	4-Me	5h	95	220–222	219–220 ref. 51
9	4Cl	4-OCH_3_	5i	94	182–184	180–183 ref. 62
10	4Cl	2,4-Dichloro	5j	92	264–66	—

a Reaction conditions: synthesis of 1,2,4,5-tetrasubstituted imidazole derivatives using benzil (1.0 mmol), aldehyde (1.0 mmol), aniline (1.0 mmol), NH_4_OAc (3.0 mmol), ZSM-11 zeolite catalyst of time 48 h (0.05 g).

b Isolated yield in 30 minutes.

**Scheme 3:**
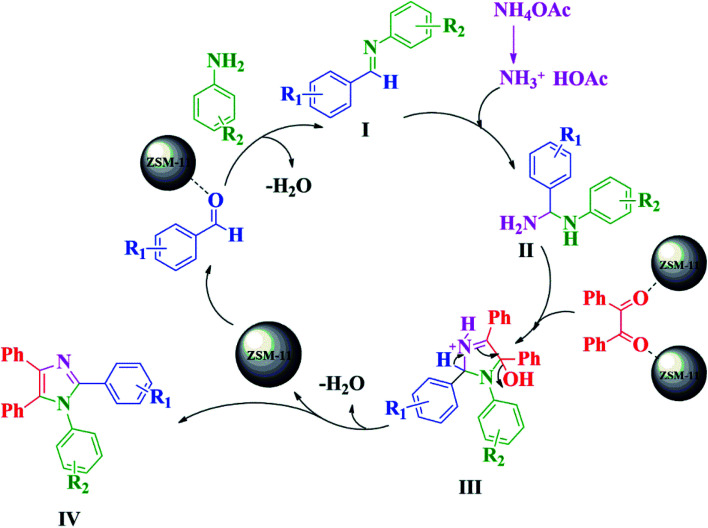
Proposed reaction mechanism for the formation of 1,2,4,5-tetrasubstituted imidazole derivatives using benzil, aldehydes, aniline, and NH_4_OAc in the presence of ZSM-11 zeolite under solvent-free conditions.

The plausible reaction mechanism for the synthesis of 1,2,4,5-tetrasubstituted imidazoles in the presence of ZSM-11 zeolite catalyst (48 h) is depicted in Scheme 3. The reaction proceeds *via* the diamine intermediate; first the aldehyde is activated by the ZSM-11 zeolite catalyst, which then reacts with the amine to create an iminium intermediate [I]. Afterward, intermediate [I] reacts with ammonia, which was formed from ammonium acetate to give diamine intermediate [II], followed by the condensation of the diamine intermediate with 1,2-diketone to give intermediate [III]. After sometime, intermediate [III] converts to 1,2,4,5-tetrasubstituted imidazole [IV] by dehydration.^11,55,56^

### Recyclability of catalysts

**Fig. 9:**
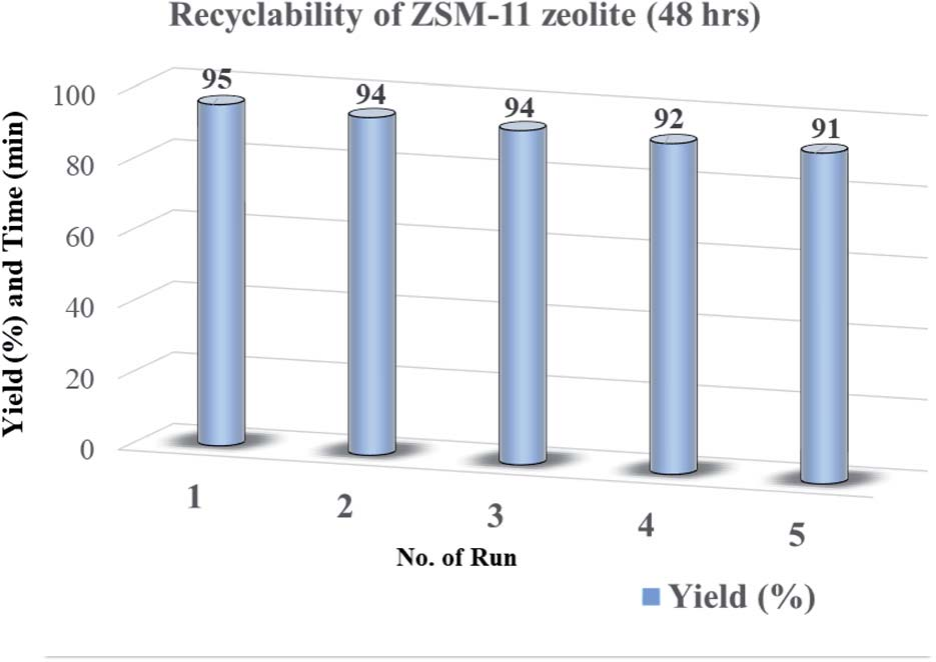
Recyclability of ZSM-11 zeolite catalyst (48 h) for the synthesis of 1,2,4,5-tetrasubstituted imidazole derivatives (5f).

To check the reusability of the catalyst is another favorable merit for the catalyst under investigation. The recycling of catalyst was performed through the reaction between benzaldehyde, benzil, aniline, and NH_4_OAc to provide tetrasubstituted imidazole derivatives (5f) under solvent-free conditions with zeolite ZSM-11 of time 48 h as a catalyst. After completion of the reaction, the separated catalyst was washed with acetone, followed by drying of the catalyst and activation in a vacuum oven for one hour at 200 °C. Then, the activated catalyst was reused in five consecutive cycles by providing the corresponding imidazole with 95%, 94%, 94%, 92%, and 91% yield; the results of which are depicted in Fig. 9. These studies proved that the current catalyst is highly reusable without a considerable reduction in the catalytic activity. After the fifth cycle, the recycled catalyst was recovered and characterized by using powder XRD and FT-IR analysis. The diffraction peaks at 2*θ*° = 8.06, 9.01, 14.95, 23.30, 24.01, and 45.4 (Fig. 1e) were consistent with the fresh catalyst (Fig. 1d), and the absorption bands at 400, 442, 547, 792, 1062, 1250, 1475, and 1640 cm^−1^ (Fig. 2e) were similar to those in the fresh catalyst (Fig. 2d).

## Conclusion

In this study, a series of ZSM-11 zeolites have been successfully synthesized with time variation by using a simple hydrothermal method at 175 °C and tetrapropyl ammonium hydroxide was used as a structure-directing agent. The zeolites were characterized by XRD, FT-IR, FE-SEM, HR-TEM, BET, EDS, and pyridine FT-IR techniques using calibrated instruments. The obtained results show that more aluminum is inserted into the zeolite framework with increasing time, resulting in increased crystallinity, surface area, external surface area, and Lewis as well as Brønsted acid sites. Also, it was evident that the ZSM-11 zeolite of 48 h exhibited the highest crystallinity, surface area, external surface area, and acidity. Additionally, the study was extended to the synthesis of biologically active 1,2,4,5-tetrasubstituted imidazoles using ZSM-11 zeolites (18, 24, 36, and 48 h). It was found that zeolite ZSM-11 (48 h) is an efficient and reusable catalyst towards the synthesis of 1,2,4,5-tetrasubstituted imidazoles under solvent-free conditions at 110 °C. This protocol has several advantages such as being highly efficient, excellent yield, work-up simplicity, no side reactions, and short reaction times. Furthermore, the current zeolite catalyst offers several attractive features such as being non-hazardous, and recovery, and reuse for at least five consecutive cycles without a significant loss in the catalytic activity with simple purification and activation procedure.

## Author contributions

Sudarshan S. Dipake-conducting experiments, writing the original draft. Vijayanand D. Ingale-investigation, Sonali A. Korde-investigation. Machhindra K. Lande-review and editing. Anjali S. Rajbhoj-review and editing. Suresh T. Gaikwad-writing, review, and editing.

## Conflicts of interest

The authors declare that there is no conflict of interest regarding the publication of this paper.

## Supplementary Material

RA-012-D1RA07984K-s001
