# Supplementary material for: An efficient green protocol for the synthesis of 1,2,4,5-tetrasubstituted imidazoles in the presence of ZSM-11 zeolite as a reusable catalyst
Source: RSC Adv. 2022 Feb 2;12(7):4358–69. doi: 10.1039/d1ra07984k (PMC8981000; doi:10.1039/d1ra07984k)
Supplement: RA-012-D1RA07984K-s001 [file RA-012-D1RA07984K-s001.pdf]

Electronic Supplementary Material (ESI) for RSC Advances.
This journal is © The Royal Society of Chemistry 2021

# An efficient green protocol for the synthesis of 1,2,4,5-tetrasubstituted imidazoles in presence of ZSM-11 zeolite as a reusable catalyst.

Sudarshan S. Dipake, Vijayanand D. Ingale, Sonali A. Korde, Machhindra K. Lande, Anjali S. Rajbhoj, Suresh T. Gaikwad.

Department of chemistry,

Dr. Babasaheb Ambedkar Marathwada University,

Aurangabad, 431004.

Email. gaikwadsuresh12@gmail.com

---

**Abstract**

In this study, we synthesized a series of ZSM-11 zeolite catalysts by using tetrapropyl ammonium hydroxide as a structure-directing agent through a highly efficient hydrothermal method. The series of catalysts were studied by different techniques such as FT-IR, XRD, FE-SEM, HR-TEM, EDS, Pyridine-FT-IR, and BET. We focused here on the varying reaction time intervals from 18 to 48 hours to investigate the effect on catalytic activities of synthesized series of catalysts. The percentages of aluminum increased in the framework of zeolites with increasing crystallinity, surface area, mesoporous area, and acidity in the series of ZSM-11 zeolites by increasing the time from 18 to 48hrs. Then, we studied the catalytic activity of a series of ZSM-11 zeolites and found that the ZSM-11 zeolite (48 hrs) possesses higher catalytic activity towards the synthesis of 1,2,4,5-tetrasubstituted imidazoles under solvent-free conditions. The present protocol scored well in excellent yield, short reaction time, clean reaction profiles, low catalyst loading, and no tedious work-up. The catalyst (ZSM-11 zeolite 48 hrs) was recycled and reused in five runs without any considerable loss of activity and product yield.

**Keywords:** Hydrothermal method, ZSM-11 zeolite, Multicomponent reaction, Solvent-free condition, 1,2,4,5-tetrasubstituted imidazole derivatives.

---

## Supplementary data information

### Preparation of Catalyst

ZSM-11 catalyst was prepared via hydrothermal pathway as shown in Scheme 1. At first, 23 ml of tetraethyl orthosilicate (TEOS) as a silica source, 20 ml of distilled deionized water (DI), and 20 ml of alcohol were mixed and stirred vigorously for one hour to form a silicate solution.

In another beaker, 1.73 gm of aluminum nitrate (Al (NO3)3) was dissolved in 20 ml of distilled deionized water with stirring at room temperature. Then, 27 ml of tetra propyl ammonium hydroxide (TPAOH) base as a structure-directing agent as well as a base were mixed dropwise and stirred vigorously for one hour at room temperature for maintaining pH 12 to form alumina solution. Finally, two separate solutions were mixed dropwise together with constant vigorous stirring for two hours to obtain a transparent viscous gel. Then, the resulting mixture was transferred in a cylindrical Teflon-lined stainless-steel autoclave for hydrothermal treatment at 175°C for 18 hrs. The solid product was separated by filtration and washed repeatedly with distilled deionized water followed by drying in an oven at 120°C for 2 hrs and finally, the product was calcined at 550°C for 5 hours in a muffle furnace to remove the template. We applied the same procedure for the batch of the diverse time such as 24, 36, and 48 hours. For comparison, all the ZSM-11 zeolites were characterized by different analysis techniques and then utilized in the synthesis of 1,2,4,5-tetrasubstituted imidazole derivatives.

**General procedure for the synthesis of 1,2,4,5-tetrasubstitutedimidazole derivatives**

All the ZSM-11 zeolites were activated by heating at 550°C for 5 hrs before placing them into reaction mass.

In a typical reaction, suspension of benzil (1 mmol), aldehyde (1 mmol), aniline (1 mmol), and ammonium acetate (3 mmol) were added in 50 ml round bottom flask and were heated in an oil bath at 110°C with continuous stirring for 30 minutes under the solvent-free condition in presence of ZSM-11 zeolite (0.05 gm) as a catalyst, confirmation of completion of the reaction was monitored by TLC using petroleum ether: ethyl acetate (6:4) as a solvent system. After the completion of the reaction, the reaction mass was cooled at room temperature, followed by ethanol was added to the crude product. The spent catalyst was separated from the residual reaction mixture by filtration then washed with acetone. The obtained crude product was recovered by solvent evaporation and further purified by recrystallizing in ethanol to the obtained pure product, we applied the same procedure for the synthesis of the further derivatives of 1,2,4,5-tetrasubstituted imidazoles (5a-j), The desired products were confirmed by comparison of their physical and spectral data with those of authentic samples, which is shown in **Table 4.**

**List of content;**

1. IR, $^{1}H$, $^{13}C$ NMR and Mass spectra (1,2,4,5 tetrasubstituted imidazole derivatives)
2. Characterization of ZSM-11 zeolite catalyst.

| **1. IR, $^1$H, $^{13}$C NMR and Mass spectra** (1,2,4,5 tetrasubstituted imidazole derivatives) |
| --- |
| 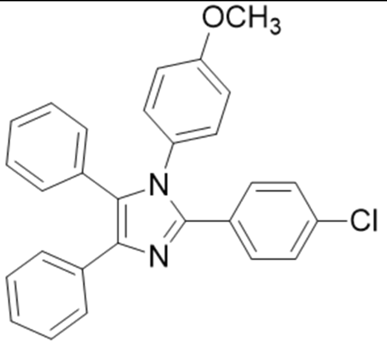 |
| **2-(4-chlorophenyl)-1-(4-methoxyphenyl)-4,5-diphenyl-1H-imidazole:** $C_{28}H_{21}ClN_2O$: **Mp.** 182-184°C: **IR (KBr, vmax/ cm$^{-1}$):** 3031, 2929, 2803, 2315, 2278, 1947, 1895, 1681, 1504, 1445: **$^1$H NMR** (400 MHz, DMSO) δ 7.47 (d, 2H), 7.42 – 7.36 (m, 4H), 7.31 (d, 3H), 7.27 – 7.13 (m, 7H), 6.87 (d, 2H), 3.71 (s, 3H). **$^{13}$C NMR** (101 MHz, DMSO) δ 158.98, 145.02, 136.81, 134.30, 133.00, 131.81, 131.09, 130.35, 129.81, 129.77, 129.32, 129.02, 128.46, 128.43, 128.27, 128.13, 126.45, 126.29, 114.28, 55.26, 40.15, 39.94, 39.73, 39.52, 39.31, 39.10, 38.89: **M$^+$** 437.13. |
| 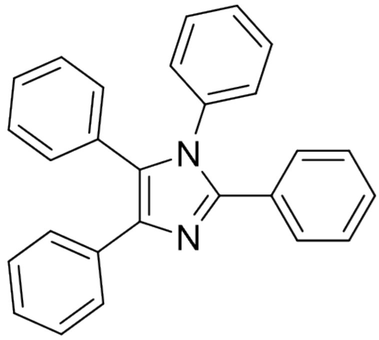 |
| **1,2,4,5-tetraphenyl-1H-imidazole: MP. IR (KBr, vmax/ cm$^{-1}$):** $C_{27}H_{20}N_2$: **Mp.** 215–217°C: 3036, 1682, 1587, 1454, 1380: **$^1$H NMR** (400 MHz, DMSO) δ 7.49 (d, 3H), 7.41 – 7.13 (m, 17H). **$^{13}$C NMR** (101 MHz, DMSO) 145.98, 136.80, 136.63, 134.37, 131.26, 131.10, 130.37, 129.11, 128.72, 128.63, 128.42, 128.38, 128.27, 128.23, 128.12, 127.03, 126.43, 126.33, 125.15, 40.15, 39.94, 39.73, 39.52, 39.31, 39.10, 38.89. **M$^+$**373.16. |
| 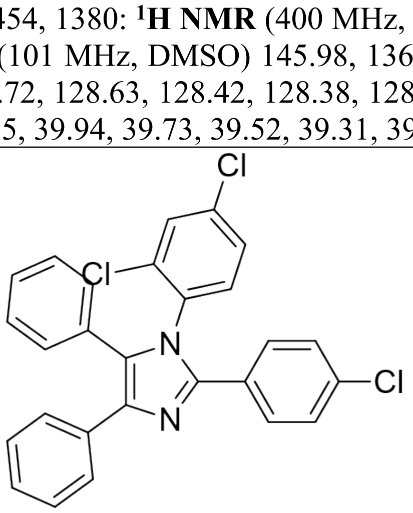 |

**2-(4-chlorophenyl)-1-(2,4-dichlorophenyl)-4,5-diphenyl-1H-imidazole:** $C_{27}H_{17}Cl_3N_2$ **: Mp.** 264-266°C: **IR (KBr, vmax/ cm$^{-1}$):** 3648, 3016, 2780, 2639, 2401, 1832, 1643, 1478, 1437: **$^1$H NMR** (400 MHz, DMSO) δ 8.10 (d, 2H), 7.58 – 7.18 (m, 15H). **$^{13}$C NMR** (101 MHz, DMSO) 144.87, 137.76, 135.46, 133.21, 131.38, 129.66, 129.24, 129.14, 129.03, 128.89, 128.67, 128.34, 127.53, 127.30, 127.07, 40.63, 40.42, 40.22, 40.01, 39.80, 39.59, 39.38. **M$^+$**475.04.

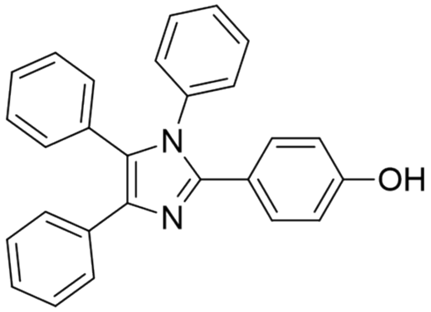


**4-(1,4,5-triphenyl-1H-imidazol-2-yl) phenol: IR (KBr, vmax/ cm$^{-1}$):** $C_{27}H_{20}N_2O$**: Mp.** 280-282°C**:** 3605, 3058, 2809, 2682, 1660, 1533, 1271, 1242: **$^1$H NMR** (400 MHz, DMSO) δ 9.67 (s, 1H), 7.48 (d, 2H), 7.34 – 7.12 (m, 15H), 6.65 (d, 2H). **$^{13}$C NMR** (101 MHz, DMSO) 158.04, 146.90, 137.33, 136.88, 135.06, 131.59, 131.09, 131.03, 130.23, 129.53, 129.24, 129.00, 128.87, 128.72, 128.56, 126.79, 126.75, 121.73, 115.39, 40.63, 40.43, 40.22, 40.01, 39.80, 39.59, 39.38.

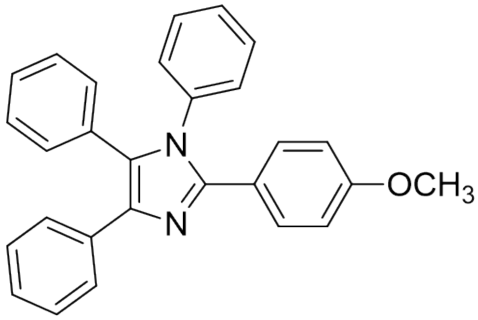


**2-(4-methoxyphenyl)-1,4,5-triphenyl-1H-imidazole:IR(KBr,vmax/ cm$^{-1}$):** $C_{28}H_{22}N_2O$: **Mp.** 182-184°C: 3059, 1603, 1375, 1295, 1248: **$^1$H NMR** (400 MHz, DMSO) δ 7.48 (d, 2H), 7.36 – 7.14 (m, 15H), 6.85 (d, 2H), 3.73 (s, 3H). **$^{13}$C NMR** (101 MHz, DMSO) δ 159.19, 145.97, 136.77, 136.52, 134.48, 131.11, 130.81, 130.51, 129.61, 129.12, 128.76, 128.66, 128.40, 128.29, 128.10, 126.31, 122.80, 113.59, 55.11, 40.15, 39.94, 39.73, 39.52, 39.31, 39.10, 38.89. **M$^+$**403.17.

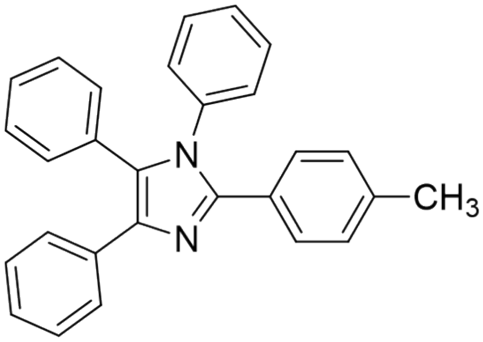


**1,4,5-triphenyl-2-(p-tolyl)-1H-imidazole: IR (KBr, vmax/ cm$^{-1}$):** $C_{28}H_{22}N_2$**: Mp.** 184-186°C: 3331, 3100, 2670, 2519, 1896, 1588, 1485, 1367: **$^1$H NMR** (400 MHz, DMSO) δ 7.49 (d, 2H), 7.34 – 7.21 (m, 14H), 7.18 (d, 1H), 7.09 (d, 2H), 2.26 (s, 3H). **$^{13}$C NMR** (101 MHz, DMSO) δ 146.09, 137.77, 136.70, 136.67, 134.43, 131.10, 131.04, 130.44, 129.08, 128.72, 128.70, 128.63, 128.56, 128.40, 128.32, 128.14, 128.10, 127.57, 126.36, 126.32,

40.15, 39.94, 39.73, 39.52, 39.31, 39.10, 38.89, 20.72. **$M^+$**387.18.

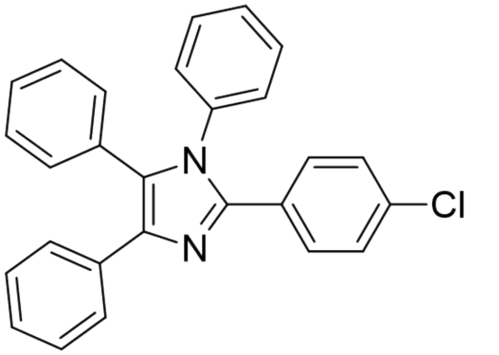


**2-(4-chlorophenyl)-1,4,5-triphenyl-1H-imidazole: IR (KBr, vmax/ $cm^{-1}$):** $C_{27}H_{19}ClN_2$ **: Mp.** 160-161°C: 3663, 1677, 1584, 1482, 1400, 1364: **$^{1}$H NMR** (400 MHz, DMSO) δ 7.49 (d, 2H), 7.40 – 7.32 (m, 7H), 7.31 – 7.22 (m, 9H), 7.18 (t, 1H). **$^{13}$C NMR** (101 MHz, DMSO) δ 145.36, 137.50, 136.90, 134.72, 133.60, 132.05, 131.58, 130.71, 130.32, 129.73, 129.69, 129.38, 129.15, 128.94, 128.76, 128.65, 127.02, 126.84, 40.65, 40.44, 40.23, 40.02, 39.81, 39.61, 39.40.

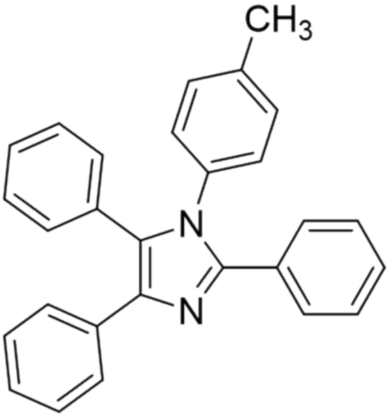


**2,4,5-triphenyl-1-(p-tolyl)-1H-imidazole: IR (KBr, vmax/ $cm^{-1}$):** $C_{28}H_{22}N_2$ **: Mp.** 284-285°C: 3530, 3027, 1801, 1589, 1501, 1437, 1384: **$^{1}$H NMR** (400 MHz, DMSO) δ 7.48 (d, 2H), 7.40 (dd, 2H), 7.29 (tt, 6H), 7.26 – 7.21 (m, 4H), 7.20 – 7.08 (m, 5H), 2.25 (s, 3H). **$^{13}$C NMR** (101 MHz, DMSO) δ 146.52, 138.55, 137.24, 134.91, 134.56, 131.80, 131.60, 130.95, 130.09, 128.92, 128.90, 128.86, 128.71, 128.61, 128.60, 126.86, 126.79, 40.64, 40.43, 40.22, 40.01, 39.80, 39.59, 39.39, 21.10. **$M^+$**387.18.

**IR**

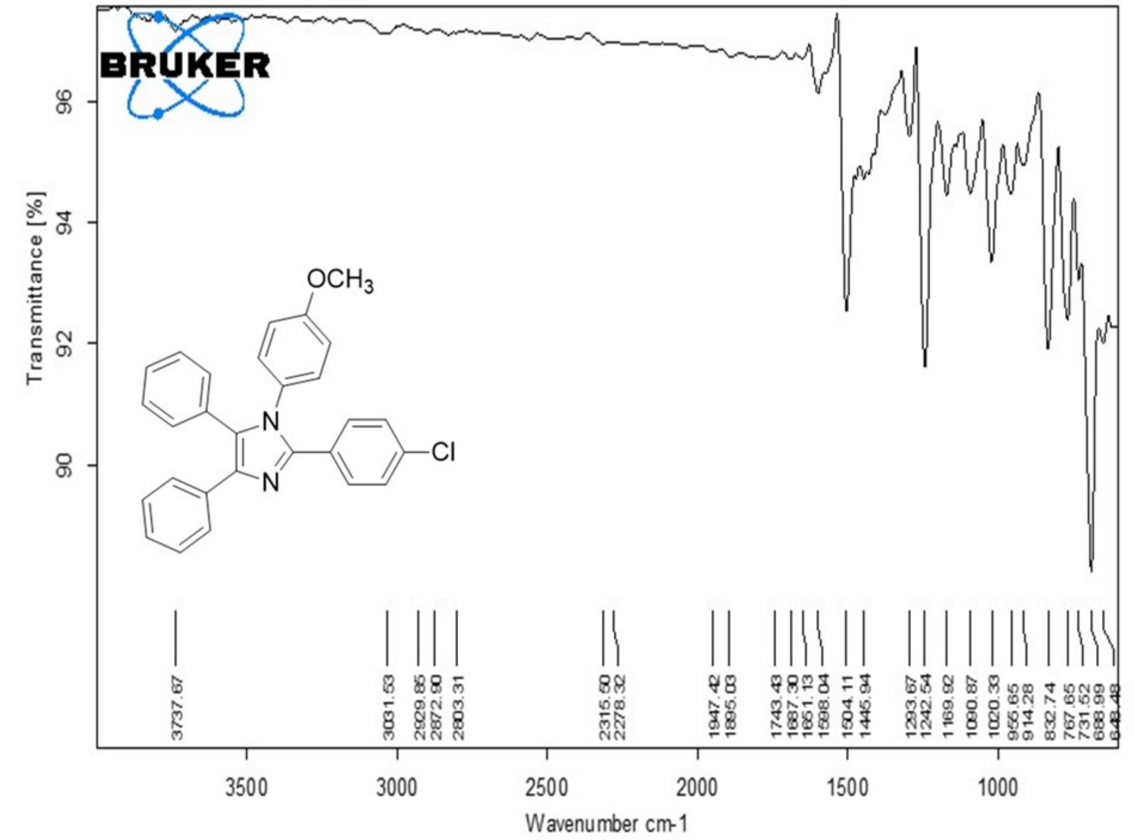


**$^{1}$H-NMR**

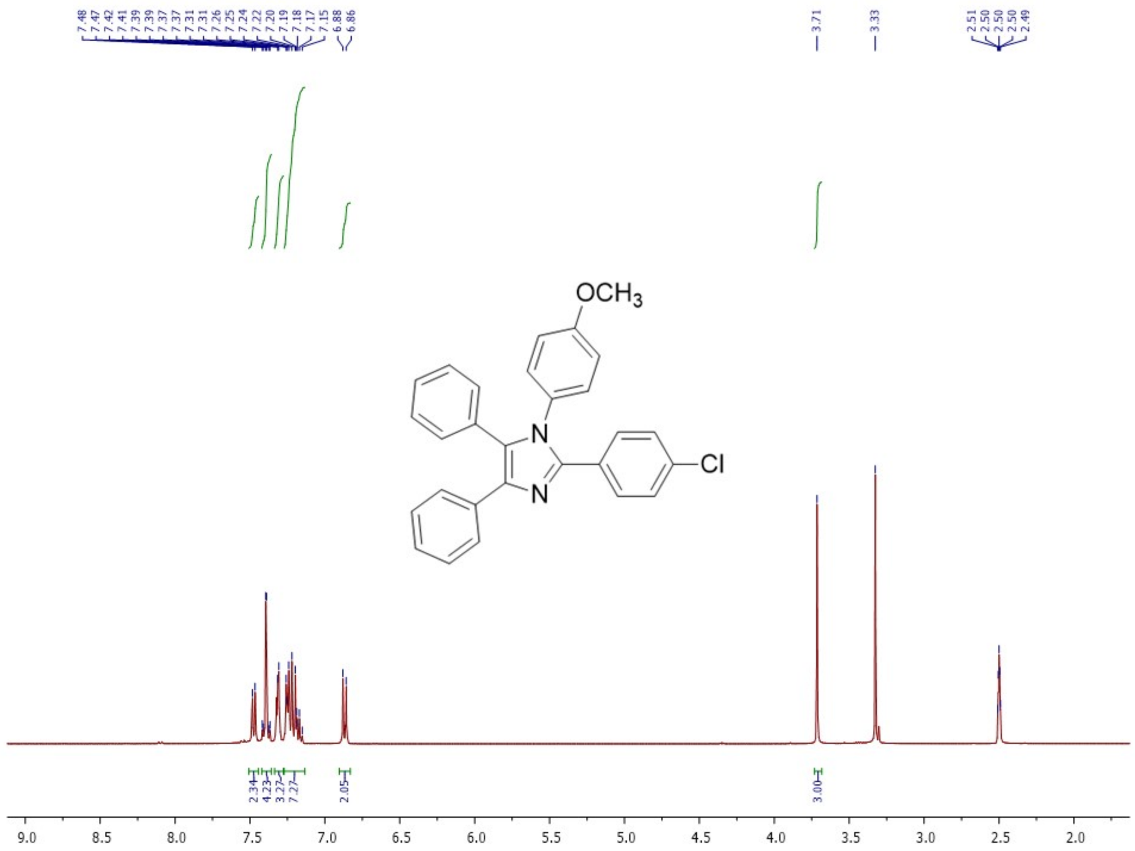


**$^{13}$C-NMR**

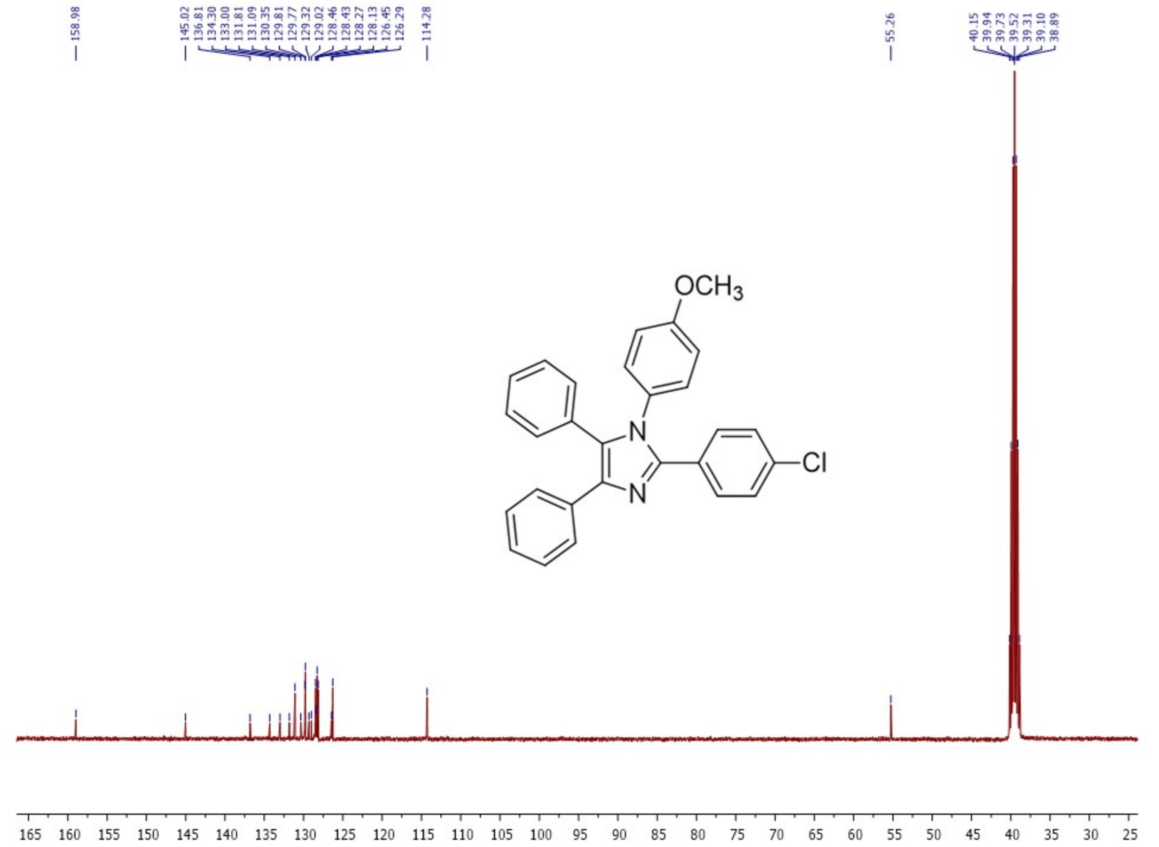
158.98
145.02
136.81
134.30
133.00
131.81
131.09
130.35
129.81
129.77
129.32
129.02
128.46
128.43
128.27
128.13
126.45
126.29
114.28
55.26
40.15
39.94
39.73
39.52
39.31
39.10
38.89
OCH3
N
N
Cl
165 160 155 150 145 140 135 130 125 120 115 110 105 100 95 90 85 80 75 70 65 60 55 50 45 40 35 30 25

**Mass**

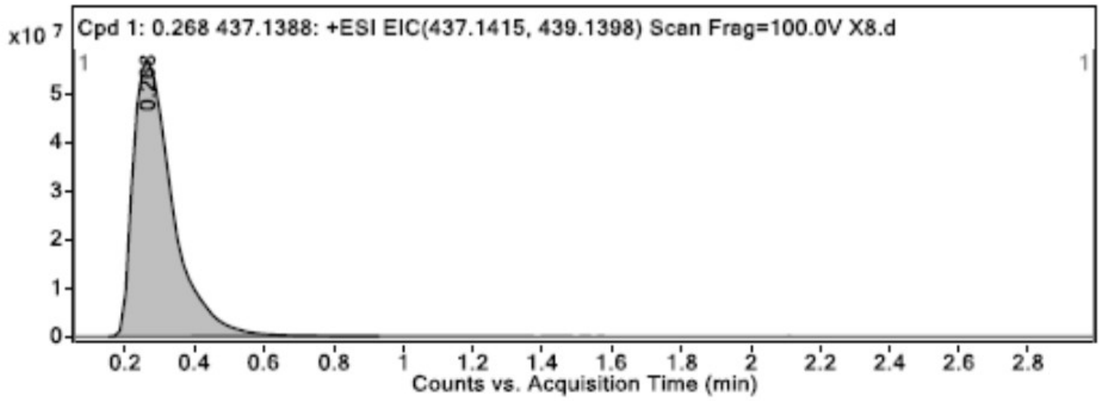
Cpd 1: 0.268 437.1388: +ESI EIC(437.1415, 439.1398) Scan Frag=100.0V X8.d
x10 7
0.268
Counts vs. Acquisition Time (min)

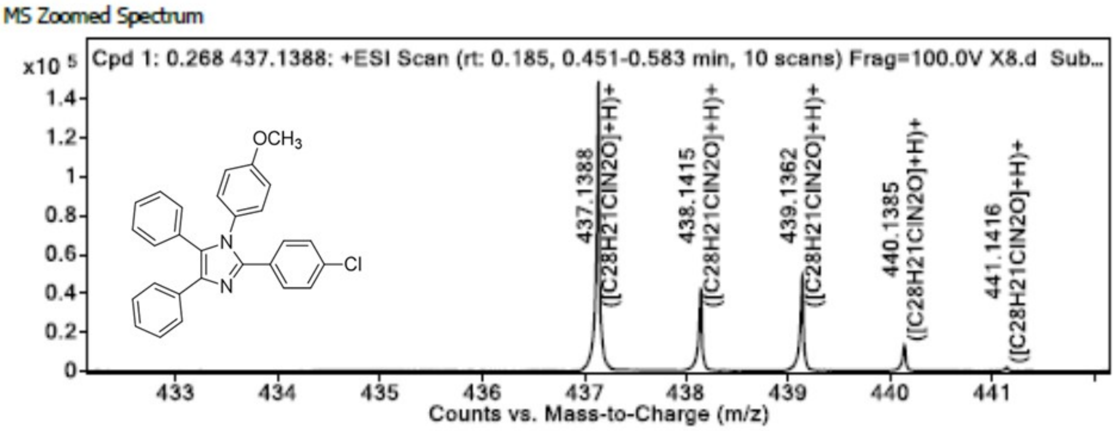
MS Zoomed Spectrum
Cpd 1: 0.268 437.1388: +ESI Scan (rt: 0.185, 0.451-0.583 min, 10 scans) Frag=100.0V X8.d Sub...
x10 5
437.1388 ([C28H21ClN2O]+H)+
438.1415 ([C28H21ClN2O]+H)+
439.1362 ([C28H21ClN2O]+H)+
440.1385 ([C28H21ClN2O]+H)+
441.1416 ([C28H21ClN2O]+H)+
OCH3
N
N
Cl
Counts vs. Mass-to-Charge (m/z)

**IR**

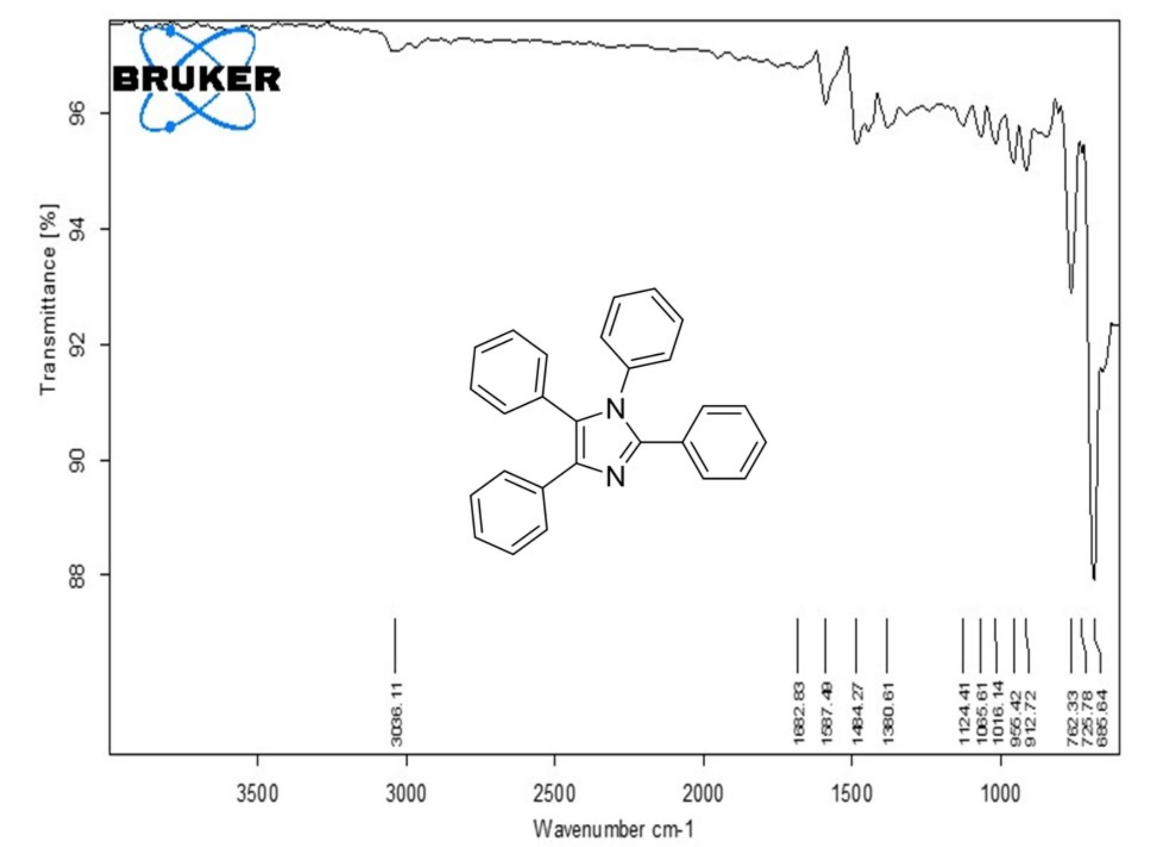


**$^{1}$H-NMR**

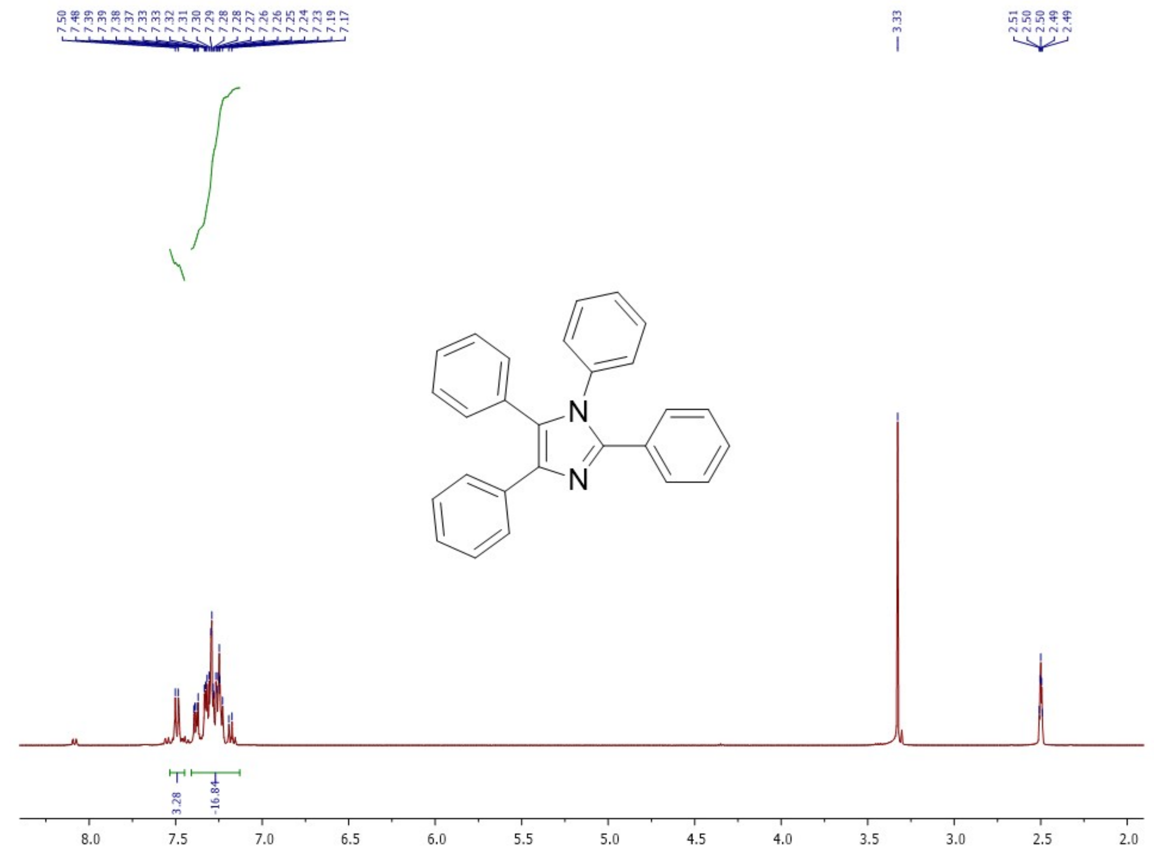


**$^{13}$C-NMR**

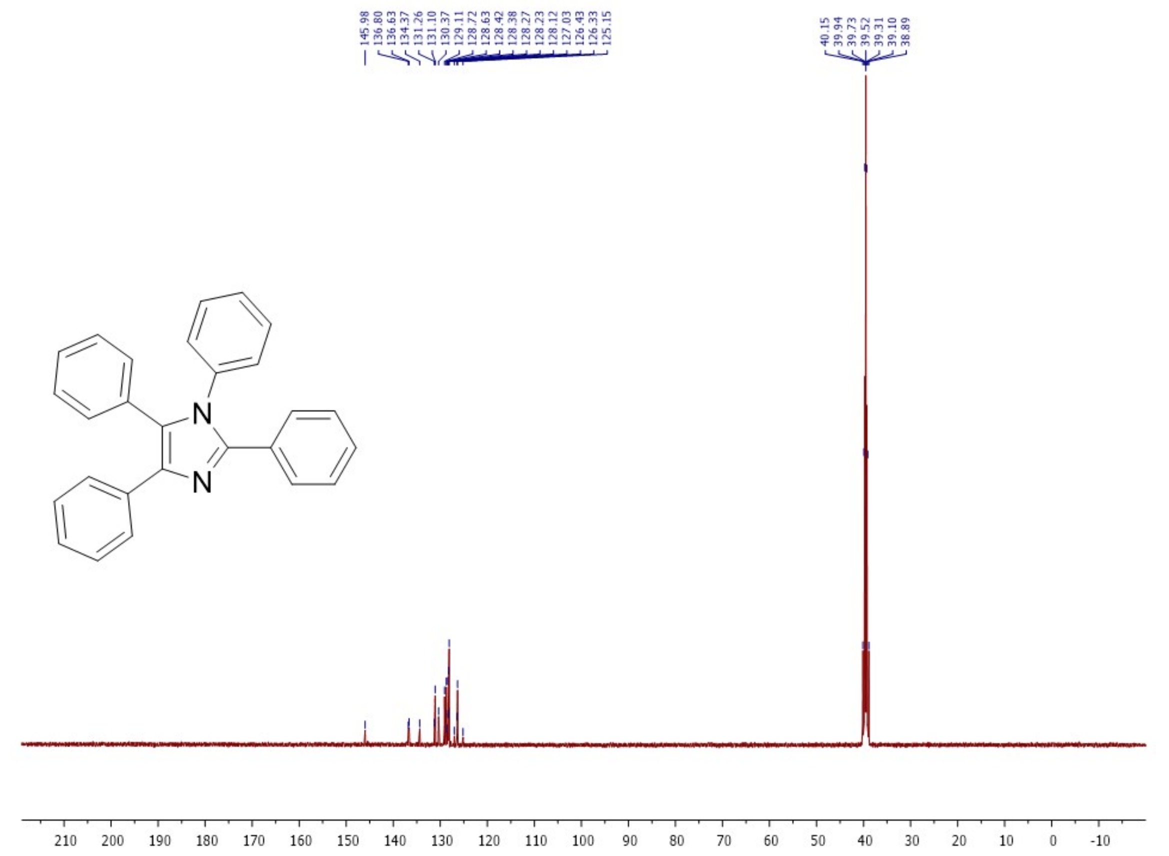
145.98
136.80
136.63
134.37
131.26
131.10
130.37
129.11
128.72
128.63
128.42
128.38
128.27
128.23
128.12
127.03
126.43
126.33
125.15
40.15
39.94
39.73
39.52
39.31
39.10
38.89
N
N
210 200 190 180 170 160 150 140 130 120 110 100 90 80 70 60 50 40 30 20 10 0 -10

**Mass**

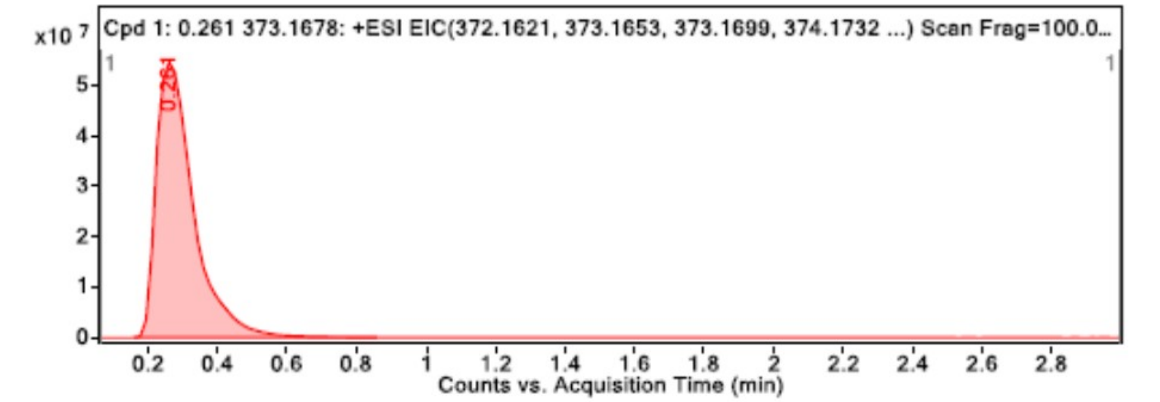
Cpd 1: 0.261 373.1678: +ESI EIC(372.1621, 373.1653, 373.1699, 374.1732 ...) Scan Frag=100.0...
x10 7
0.261
Counts vs. Acquisition Time (min)

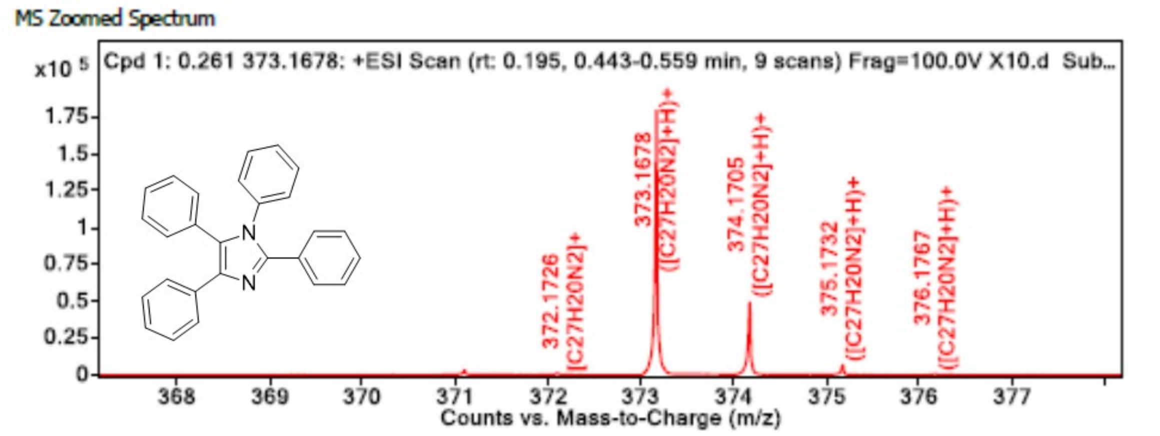
MS Zoomed Spectrum
Cpd 1: 0.261 373.1678: +ESI Scan (rt: 0.195, 0.443-0.559 min, 9 scans) Frag=100.0V X10.d Sub...
x10 5
372.1726
[C27H20N2]+
373.1678
([C27H20N2]+H)+
374.1705
([C27H20N2]+H)+
375.1732
([C27H20N2]+H)+
376.1767
([C27H20N2]+H)+
N
N
Counts vs. Mass-to-Charge (m/z)

**IR**

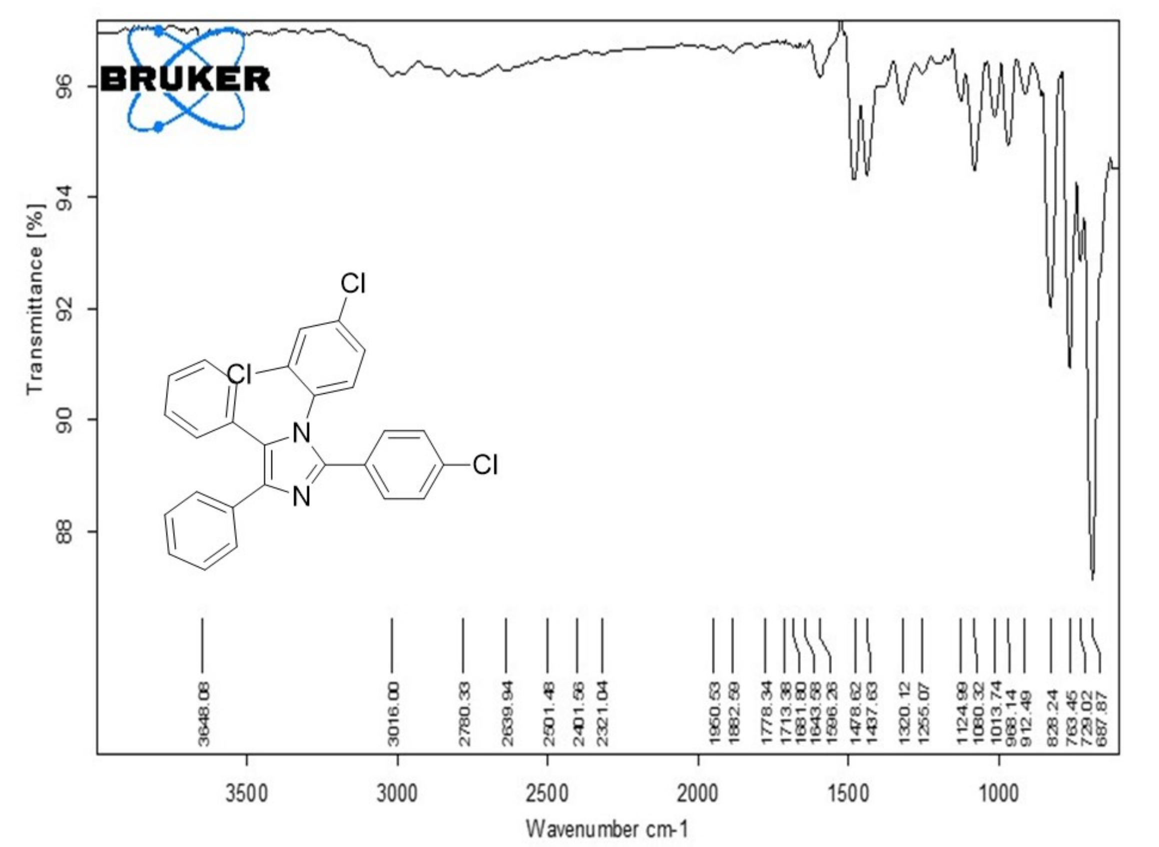


**$^{1}$H-NMR**

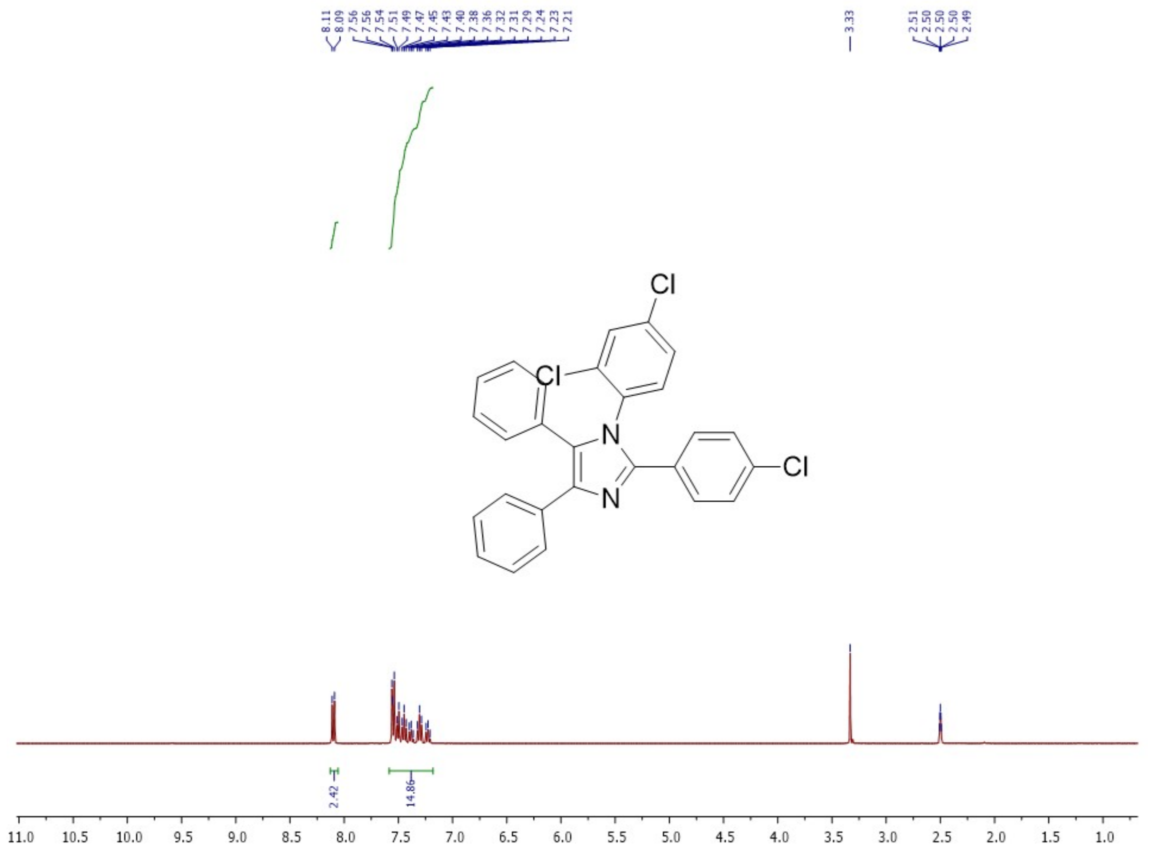


**$^{13}$C-NMR**

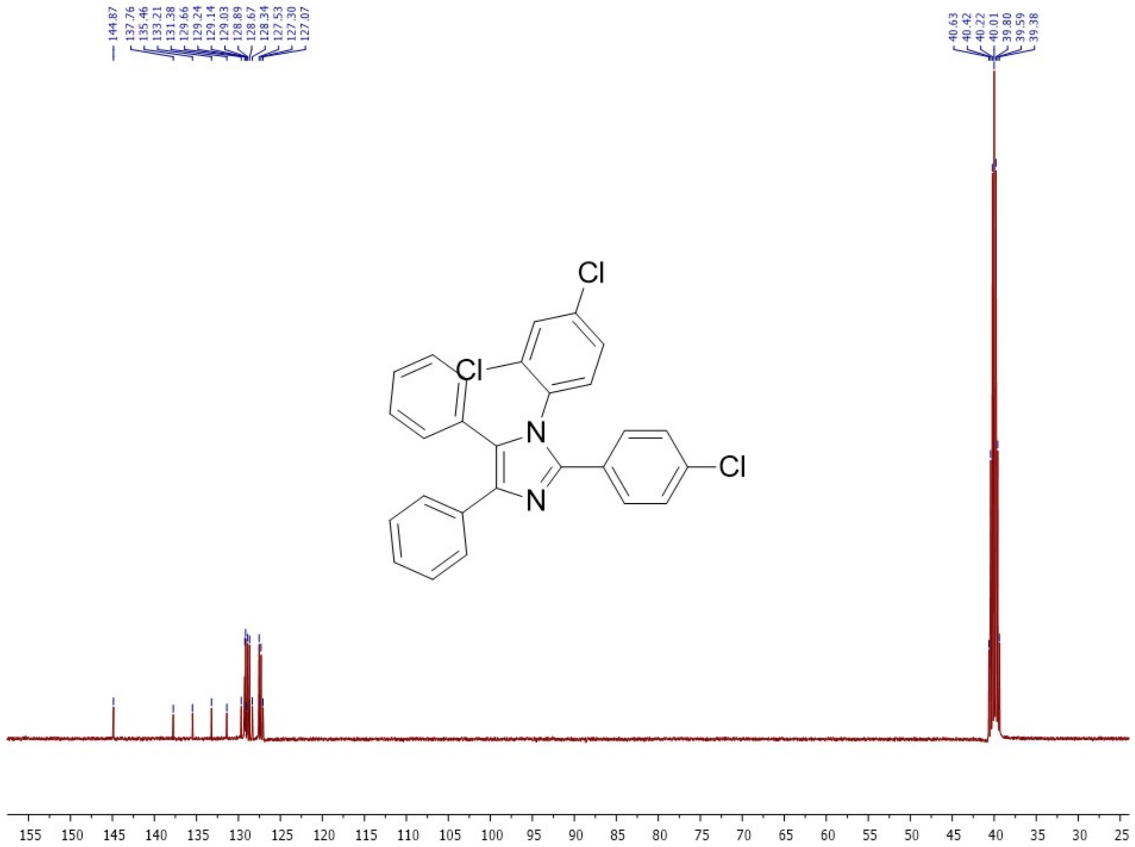


**Mass**

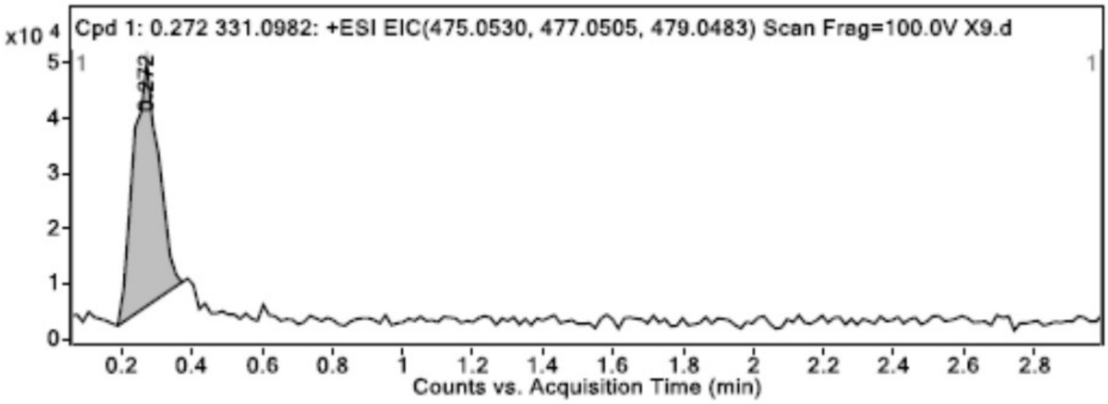


MS Zoomed Spectrum

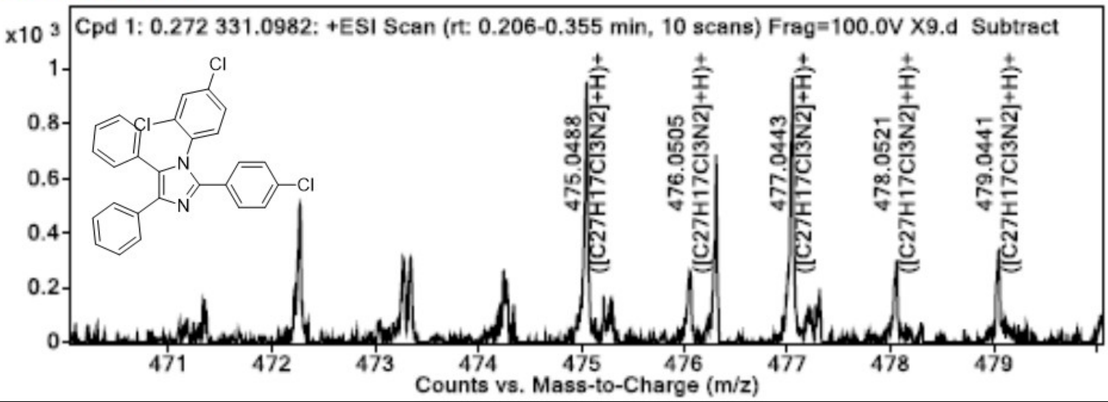


**IR**

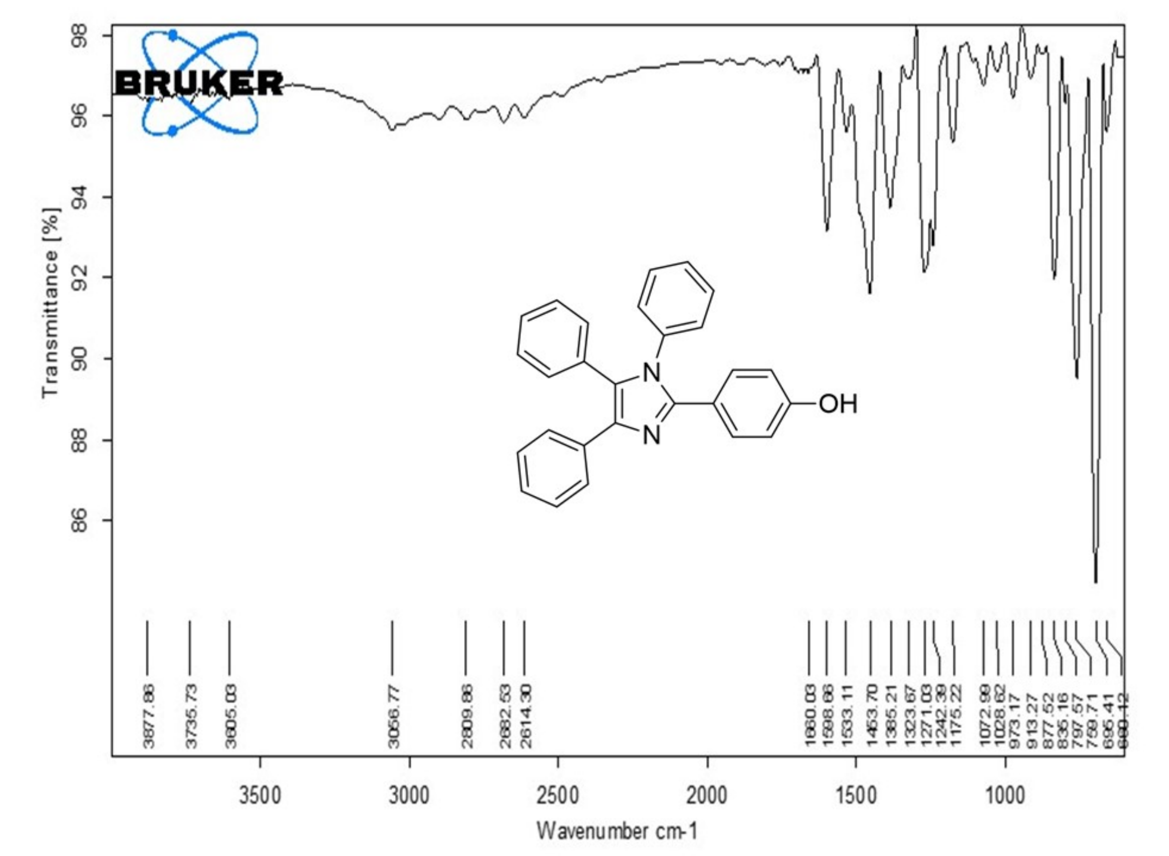


# $^{1}$H-NMR

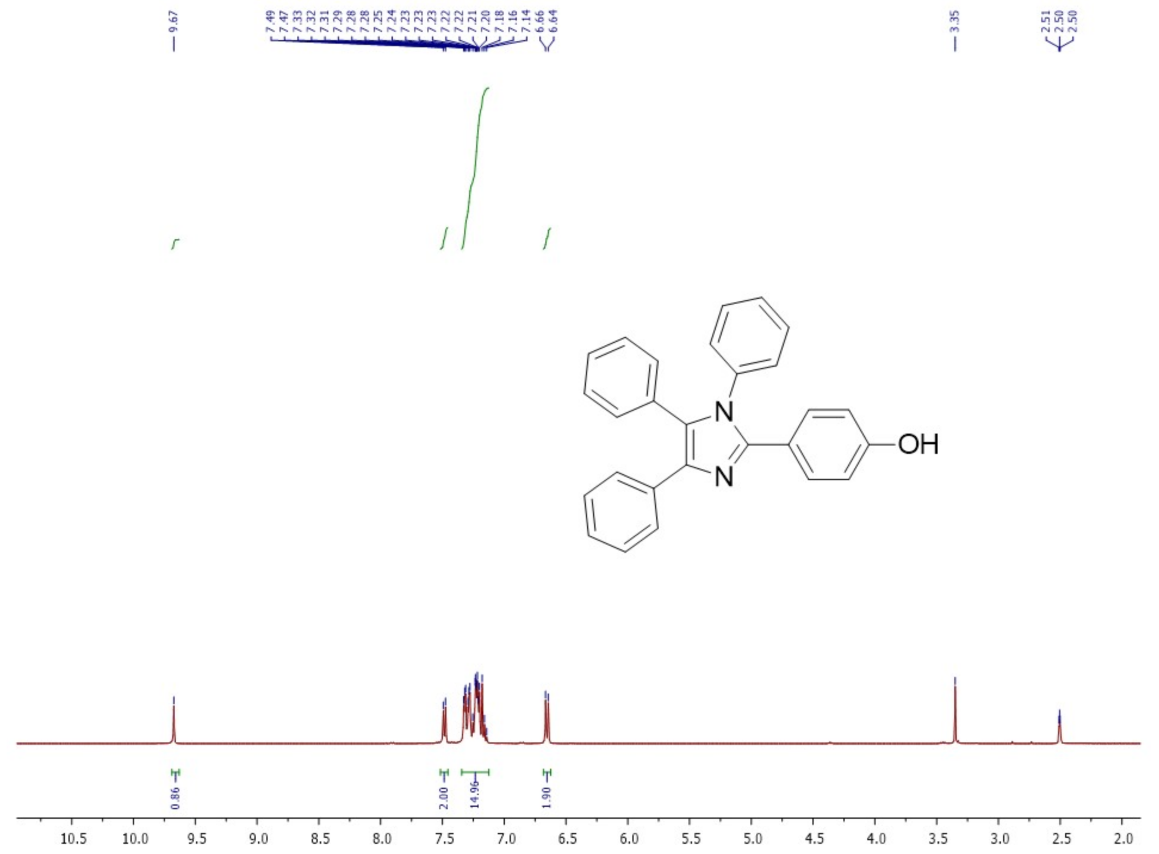


# $^{13}$C-NMR

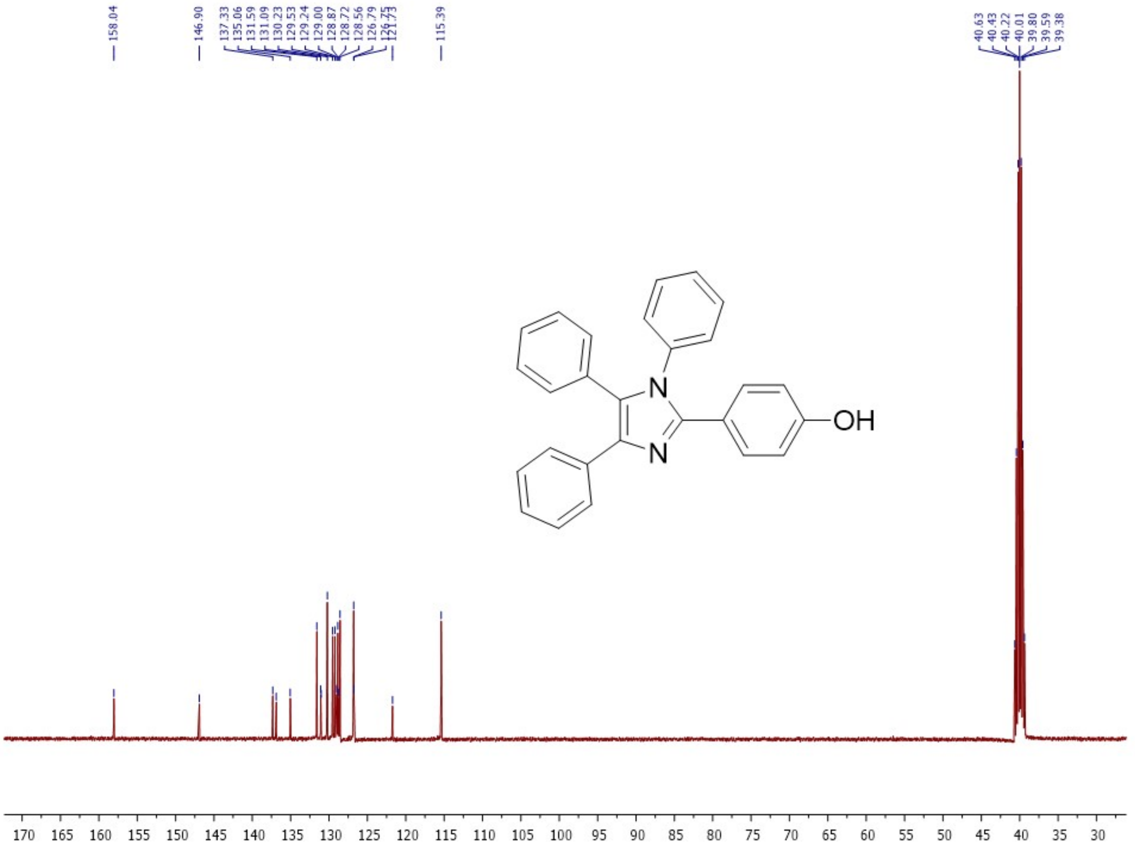
158.04
146.90
137.33
135.06
131.59
131.09
130.23
129.53
129.24
129.00
128.87
128.72
128.56
126.79
126.75
121.73
115.39
40.63
40.43
40.22
40.01
39.80
39.59
39.38
N
N
OH

**IR**

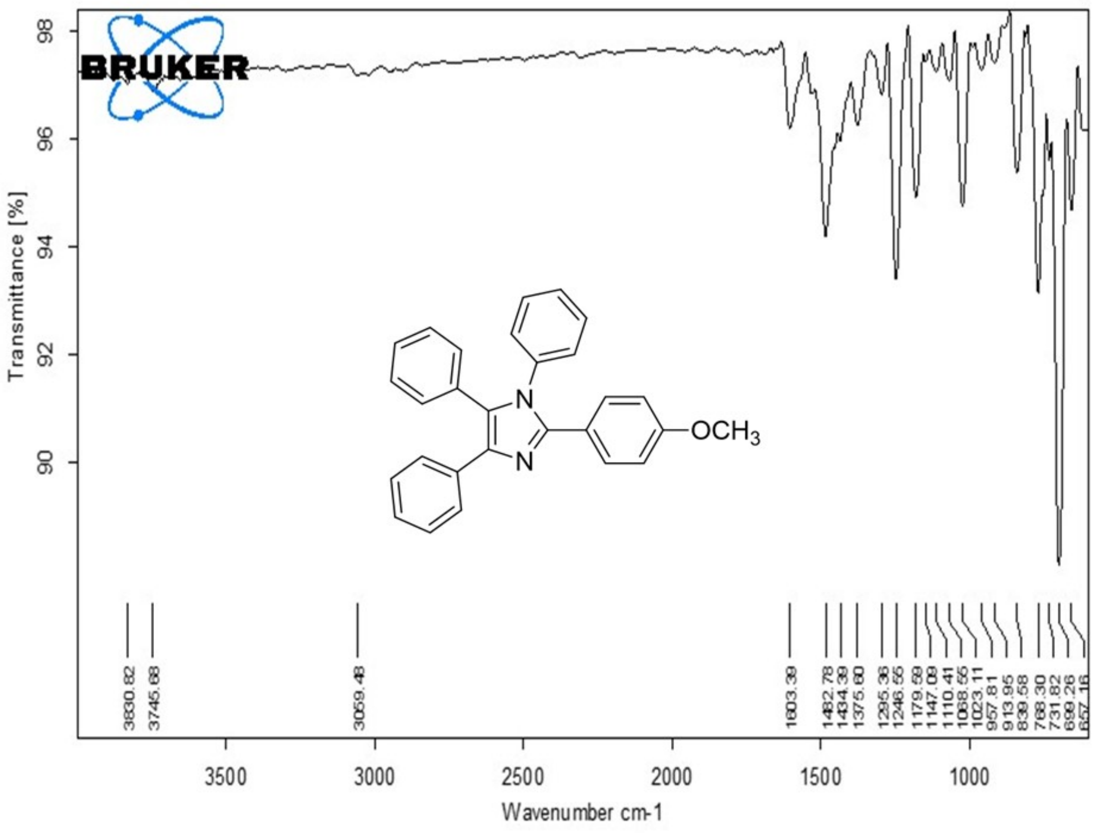
BRUKER
Transmittance [%]
Wavenumber cm-1
N
N
OCH3
3830.82
3745.68
3059.48
1603.39
1482.78
1434.39
1375.60
1295.36
1246.55
1179.59
1147.09
1110.41
1068.55
1023.11
957.81
913.95
839.58
768.30
731.82
699.26
657.16

**$^{1}$H-NMR**

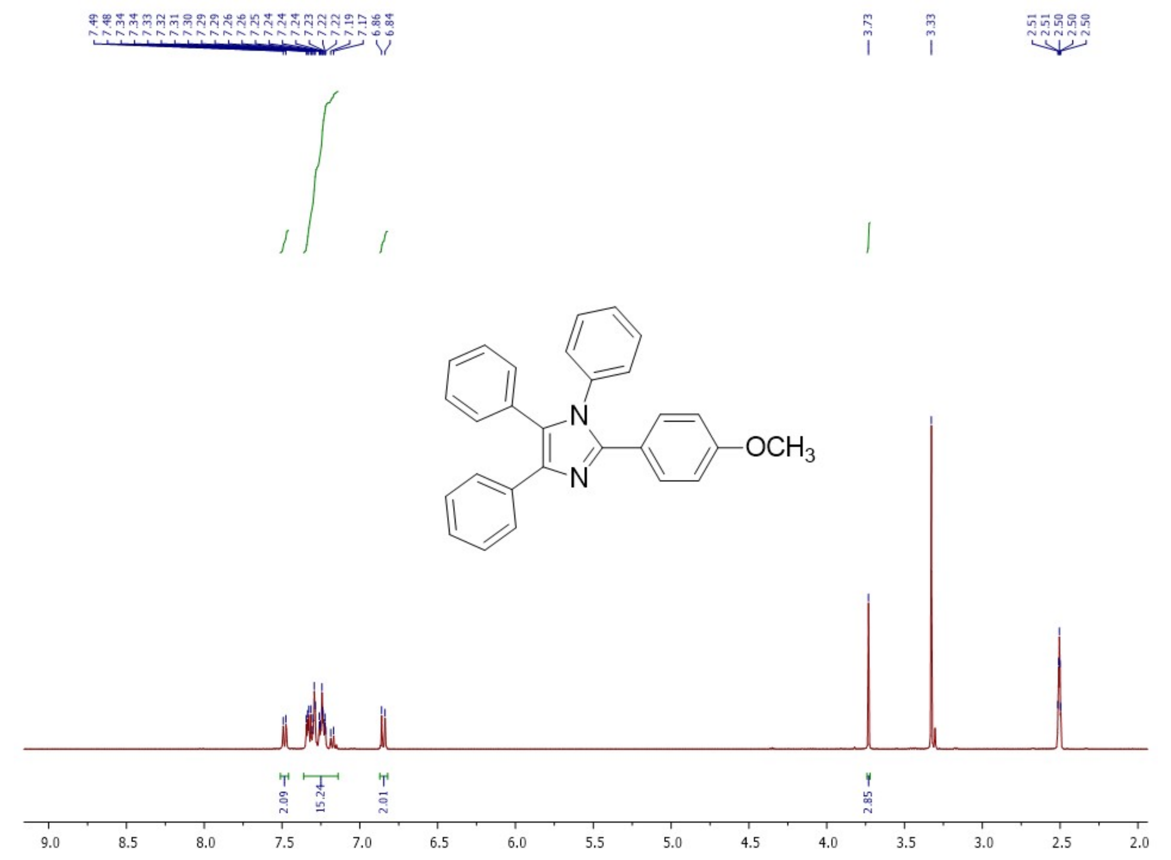
7.49 7.48 7.34 7.34 7.33 7.32 7.31 7.30 7.29 7.29 7.26 7.25 7.24 7.24 7.24 7.23 7.22 7.22 7.19 7.17 6.86 6.84
3.73
3.33
2.51 2.51 2.50 2.50 2.50
N
N
OCH3
2.09
15.24
2.01
2.85
9.0 8.5 8.0 7.5 7.0 6.5 6.0 5.5 5.0 4.5 4.0 3.5 3.0 2.5 2.0

## $^{13}$C-NMR

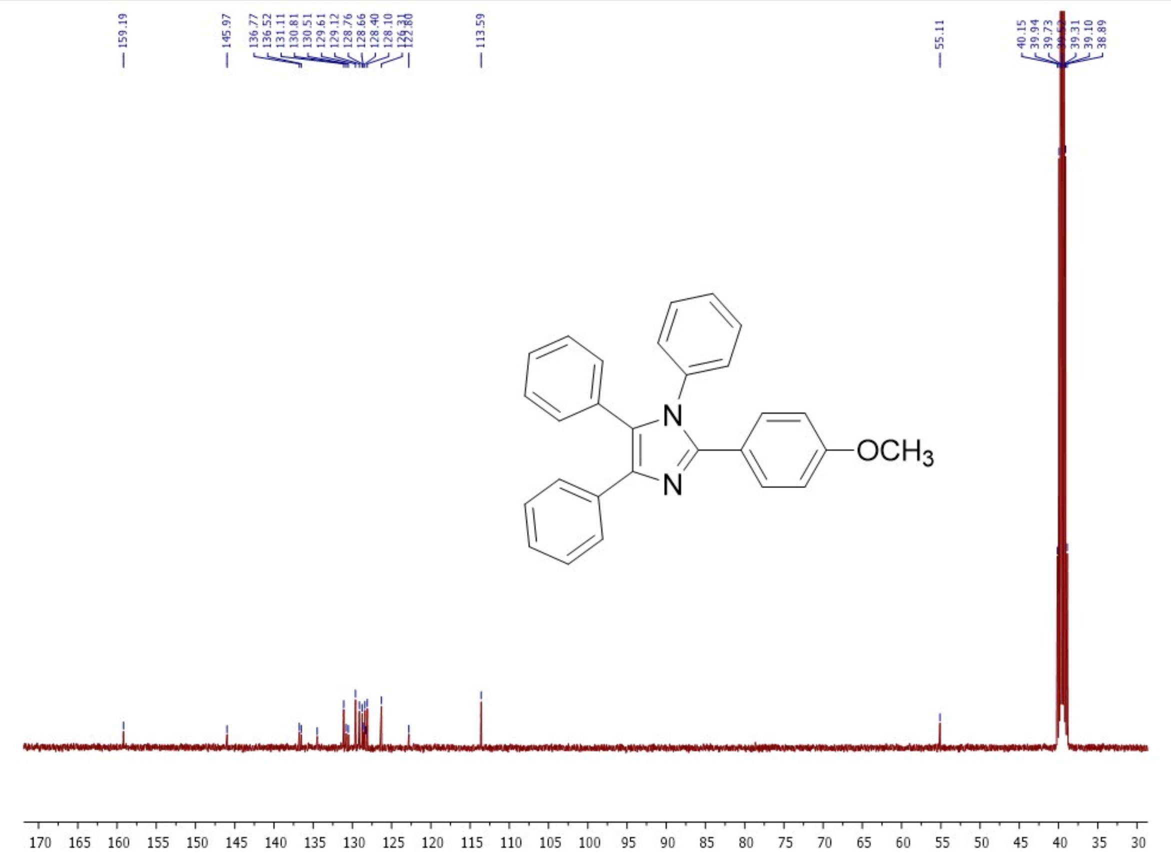
159.19
145.97
136.77 136.52 131.11 130.81 130.51 129.61 129.12 128.76 128.66 128.40 128.10 126.31 122.80
113.59
55.11
40.15 39.94 39.73 39.31 39.10 38.89
N
N
OCH3
170 165 160 155 150 145 140 135 130 125 120 115 110 105 100 95 90 85 80 75 70 65 60 55 50 45 40 35 30

## Mass

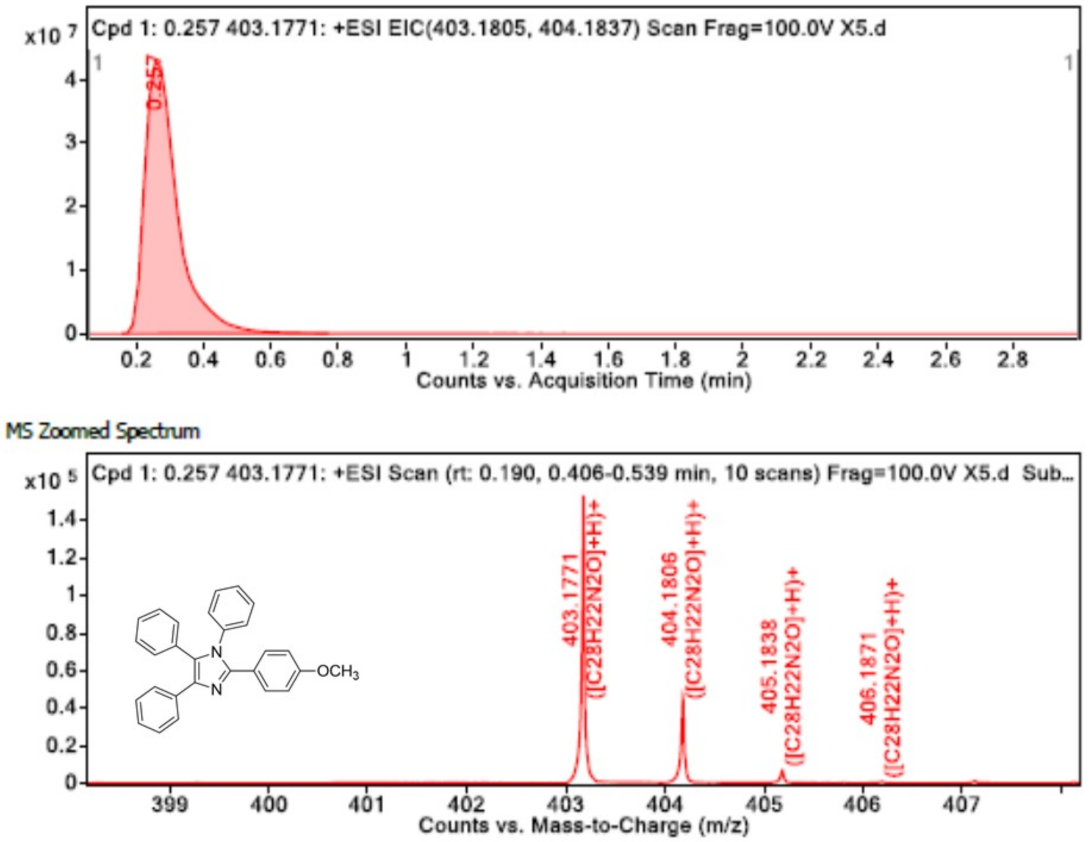
x10 7
Cpd 1: 0.257 403.1771: +ESI EIC(403.1805, 404.1837) Scan Frag=100.0V X5.d
0.257
Counts vs. Acquisition Time (min)
MS Zoomed Spectrum
x10 5
Cpd 1: 0.257 403.1771: +ESI Scan (rt: 0.190, 0.406-0.539 min, 10 scans) Frag=100.0V X5.d Sub...
403.1771 ([C28H22N2O]+H)+
404.1806 ([C28H22N2O]+H)+
405.1838 ([C28H22N2O]+H)+
406.1871 ([C28H22N2O]+H)+
N
N
OCH3
Counts vs. Mass-to-Charge (m/z)

**IR**

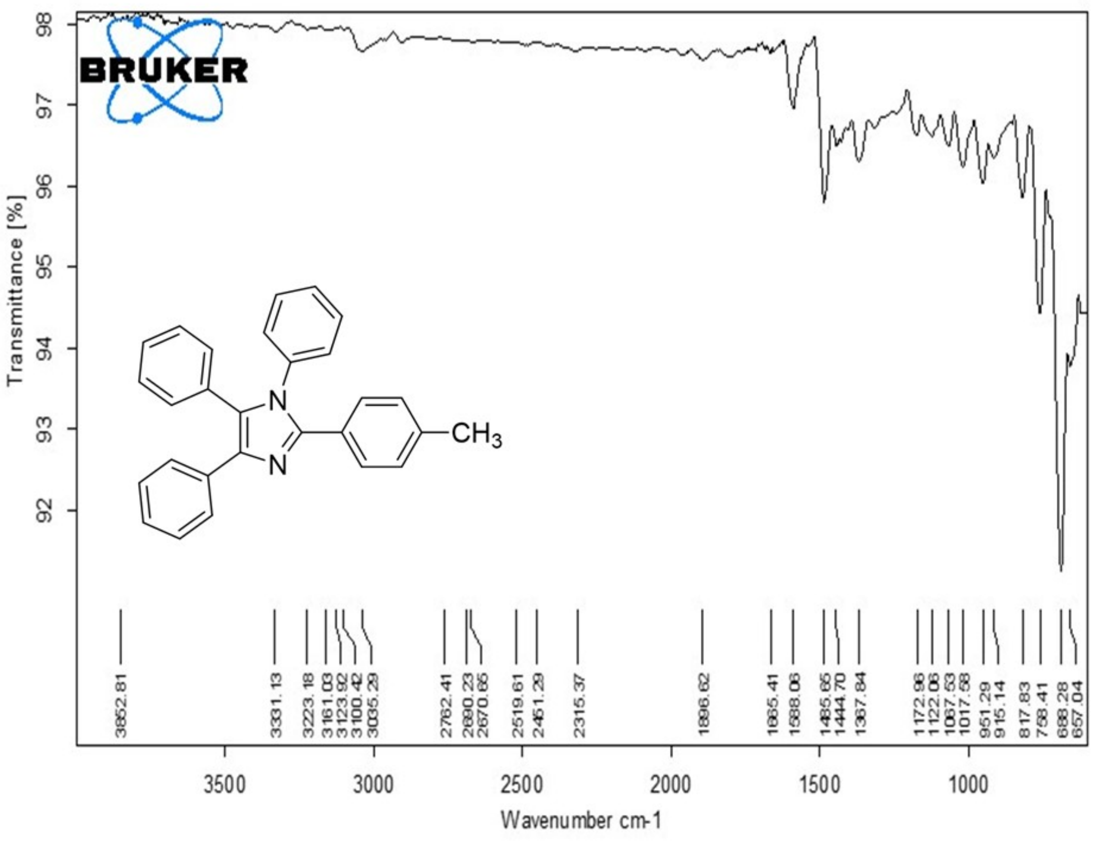
BRUKER
Transmittance [%]
N
N
CH3
3852.81
3331.13
3223.18
3161.03
3123.92
3100.42
3035.29
2762.41
2690.23
2670.65
2519.61
2451.29
2315.37
1896.62
1665.41
1588.08
1485.65
1444.70
1367.84
1172.98
1122.08
1067.53
1017.58
951.29
915.14
817.83
758.41
688.28
657.04
Wavenumber cm-1

**$^{1}$H-NMR**

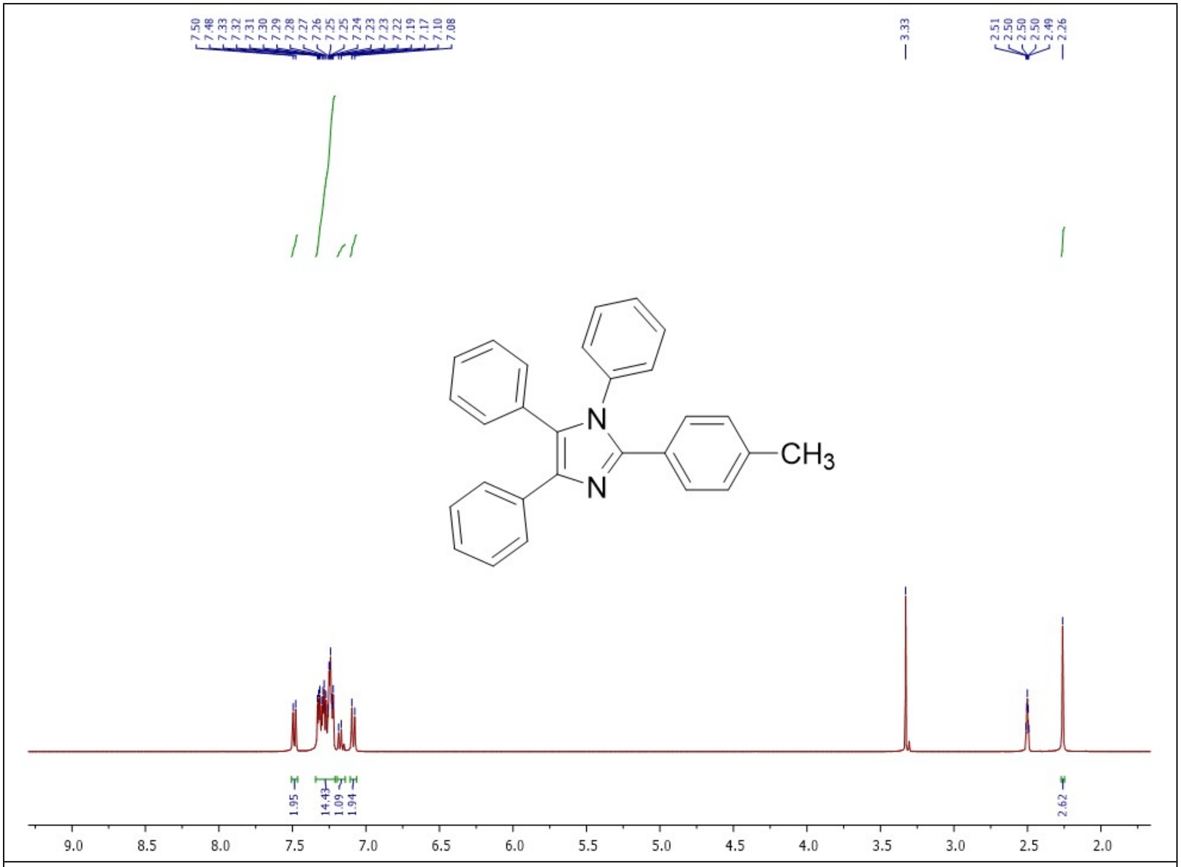
7.50 7.48 7.33 7.32 7.31 7.30 7.29 7.28 7.27 7.26 7.25 7.25 7.24 7.23 7.23 7.22 7.19 7.17 7.10 7.08
3.33
2.51 2.50 2.50 2.50 2.49 2.26
N
N
CH3
1.95 14.43 1.09 1.94 2.62
9.0 8.5 8.0 7.5 7.0 6.5 6.0 5.5 5.0 4.5 4.0 3.5 3.0 2.5 2.0

## $^{13}$C-NMR

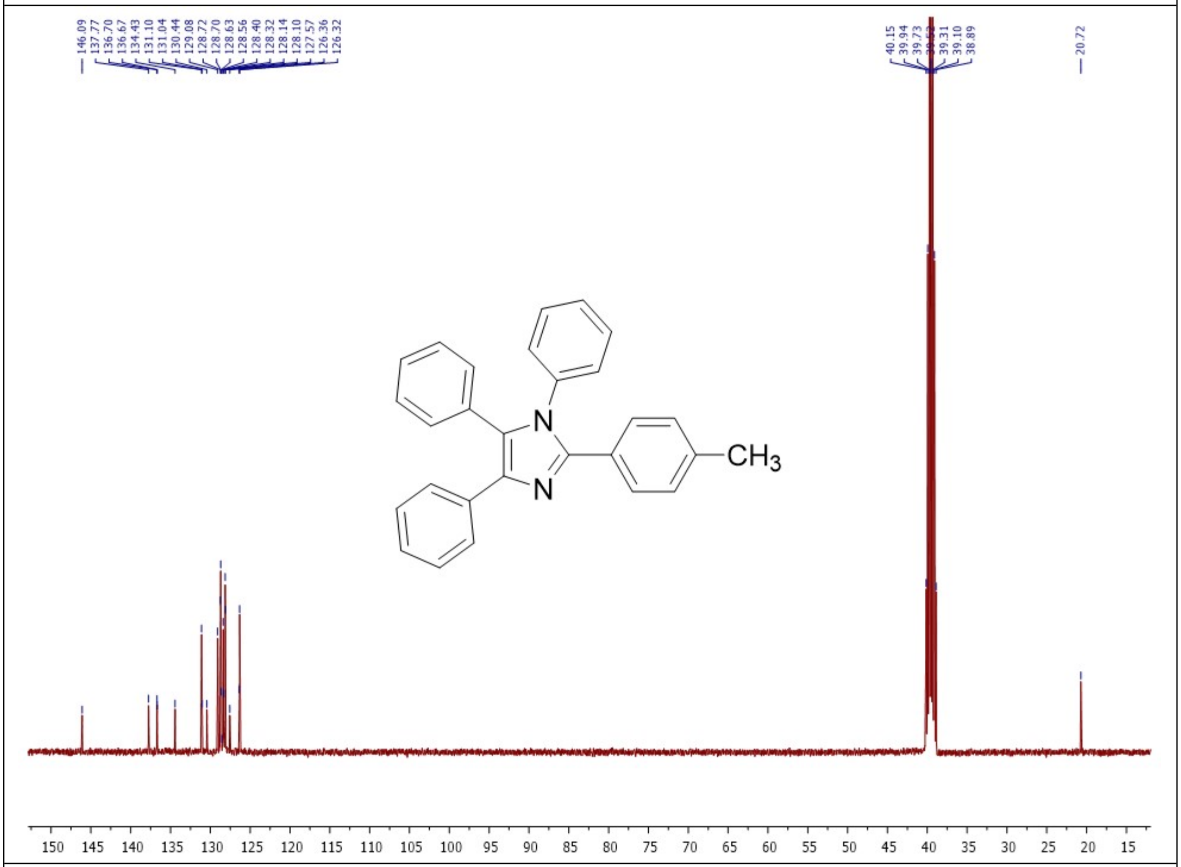
146.09 137.77 136.70 136.67 134.43 131.10 131.04 130.44 129.08 128.72 128.70 128.63 128.56 128.40 128.32 128.14 128.10 127.57 126.36 126.32
40.15 39.94 39.73 39.31 39.10 38.89
20.72
N
N
CH3
150 145 140 135 130 125 120 115 110 105 100 95 90 85 80 75 70 65 60 55 50 45 40 35 30 25 20 15

## Mass

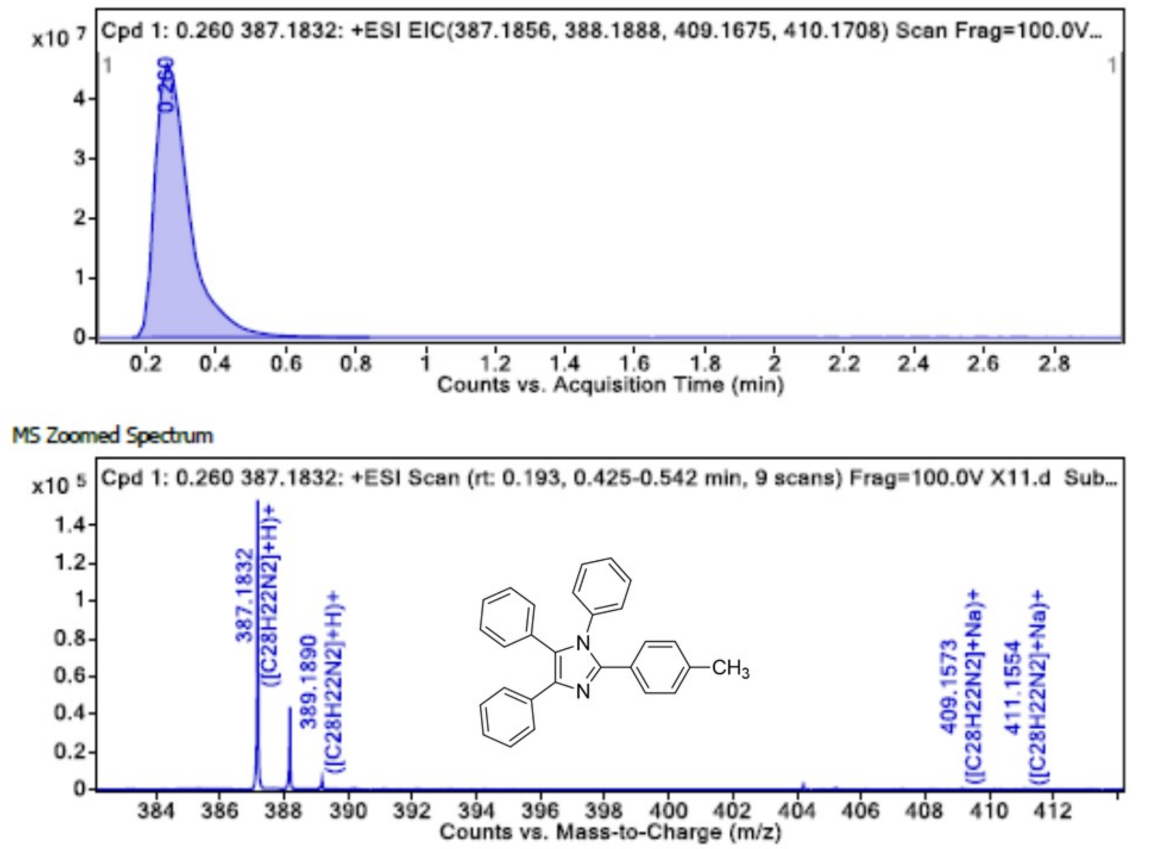
x10 7
Cpd 1: 0.260 387.1832: +ESI EIC(387.1856, 388.1888, 409.1675, 410.1708) Scan Frag=100.0V...
0.260
Counts vs. Acquisition Time (min)
MS Zoomed Spectrum
x10 5
Cpd 1: 0.260 387.1832: +ESI Scan (rt: 0.193, 0.425-0.542 min, 9 scans) Frag=100.0V X11.d Sub...
387.1832
([C28H22N2]+H)+
389.1890
([C28H22N2]+H)+
409.1573
([C28H22N2]+Na)+
411.1554
([C28H22N2]+Na)+
N
N
CH3
Counts vs. Mass-to-Charge (m/z)

**IR**

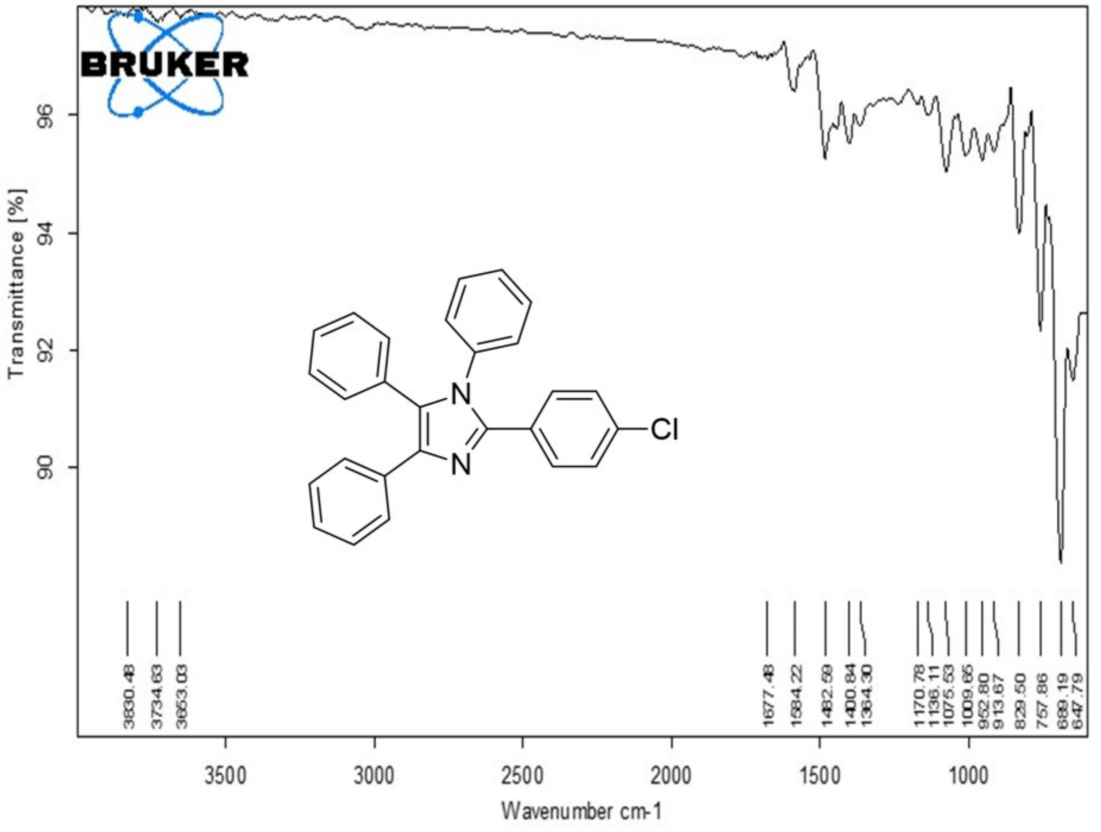
BRUKER
Transmittance [%]
N
N
Cl
3830.48
3734.63
3653.03
1677.48
1584.22
1482.59
1400.84
1364.30
1170.78
1136.11
1075.53
1009.65
952.80
913.67
829.50
757.86
689.19
647.79
Wavenumber cm-1

**$^{1}$H-NMR**

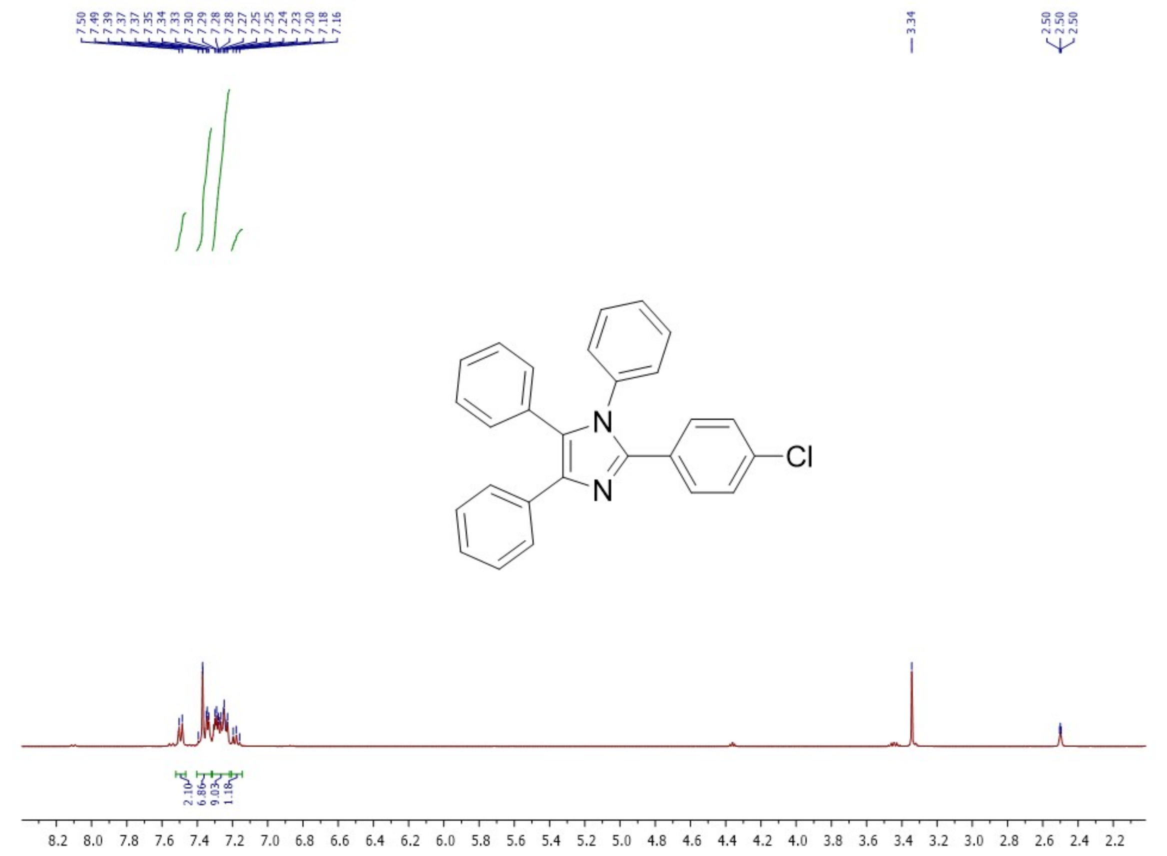
7.50 7.49 7.39 7.37 7.37 7.35 7.34 7.33 7.30 7.29 7.28 7.28 7.27 7.25 7.25 7.24 7.23 7.20 7.18 7.16
3.34
2.50 2.50 2.50
N
N
Cl
2.10 6.86 9.03 1.18
8.2 8.0 7.8 7.6 7.4 7.2 7.0 6.8 6.6 6.4 6.2 6.0 5.8 5.6 5.4 5.2 5.0 4.8 4.6 4.4 4.2 4.0 3.8 3.6 3.4 3.2 3.0 2.8 2.6 2.4 2.2

**$^{13}$C-NMR**

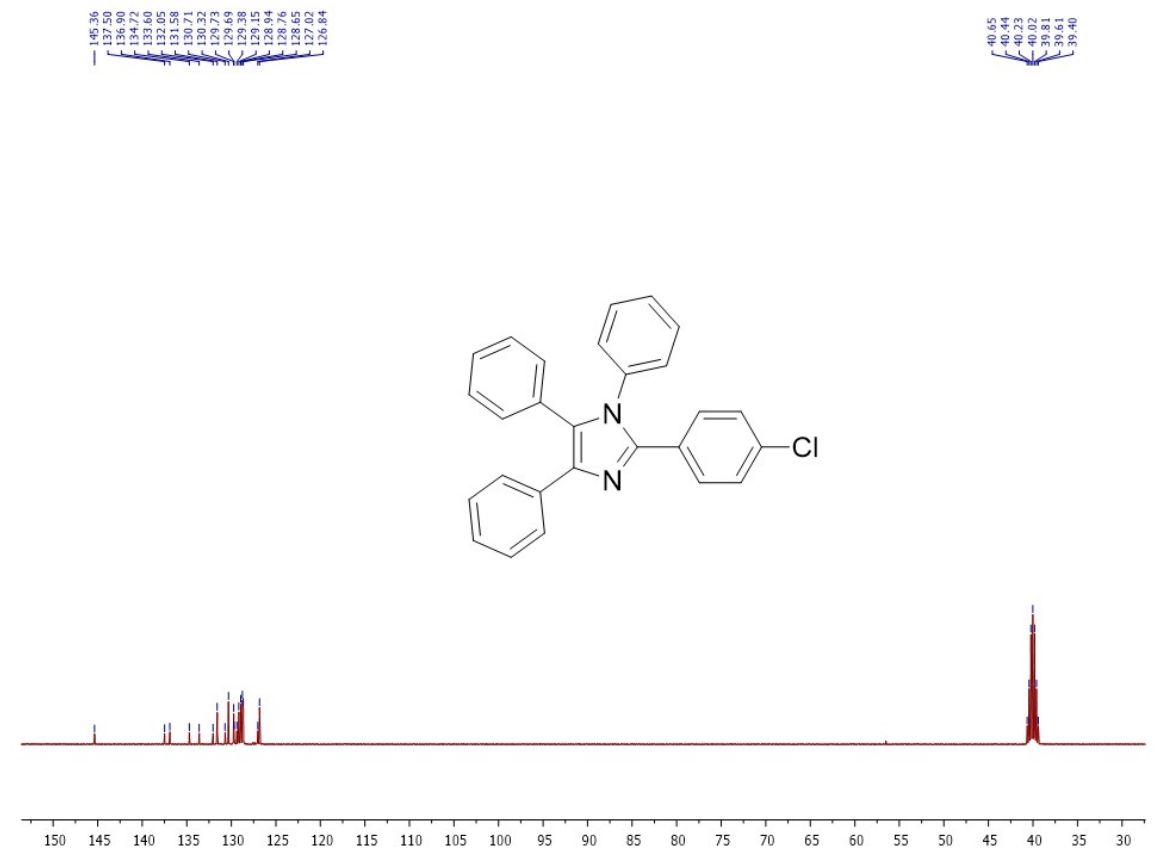
145.36 137.50 136.90 134.72 133.60 132.05 131.58 130.71 130.32 129.73 129.69 129.38 129.15 128.94 128.76 128.65 127.02 126.84
40.65 40.44 40.23 40.02 39.81 39.61 39.40
N
N
Cl
150 145 140 135 130 125 120 115 110 105 100 95 90 85 80 75 70 65 60 55 50 45 40 35 30

**IR**

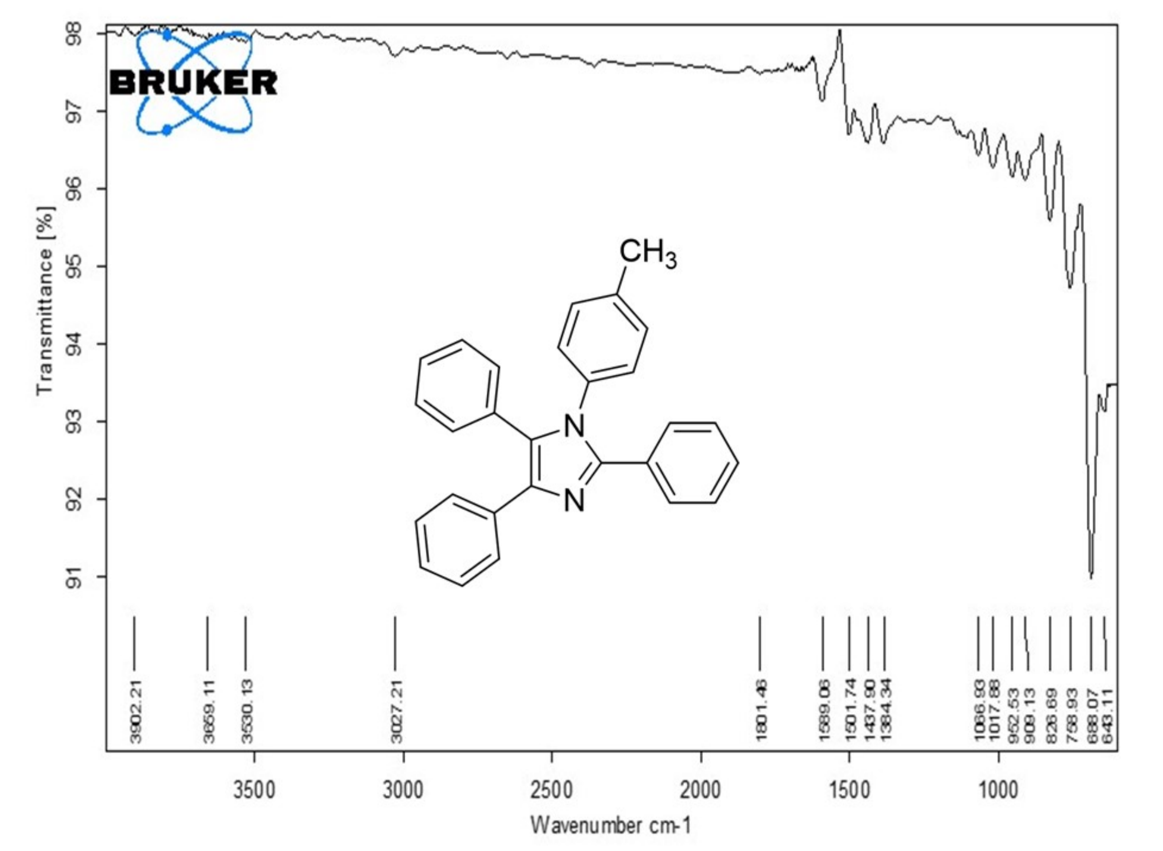


**$^{1}$H-NMR**

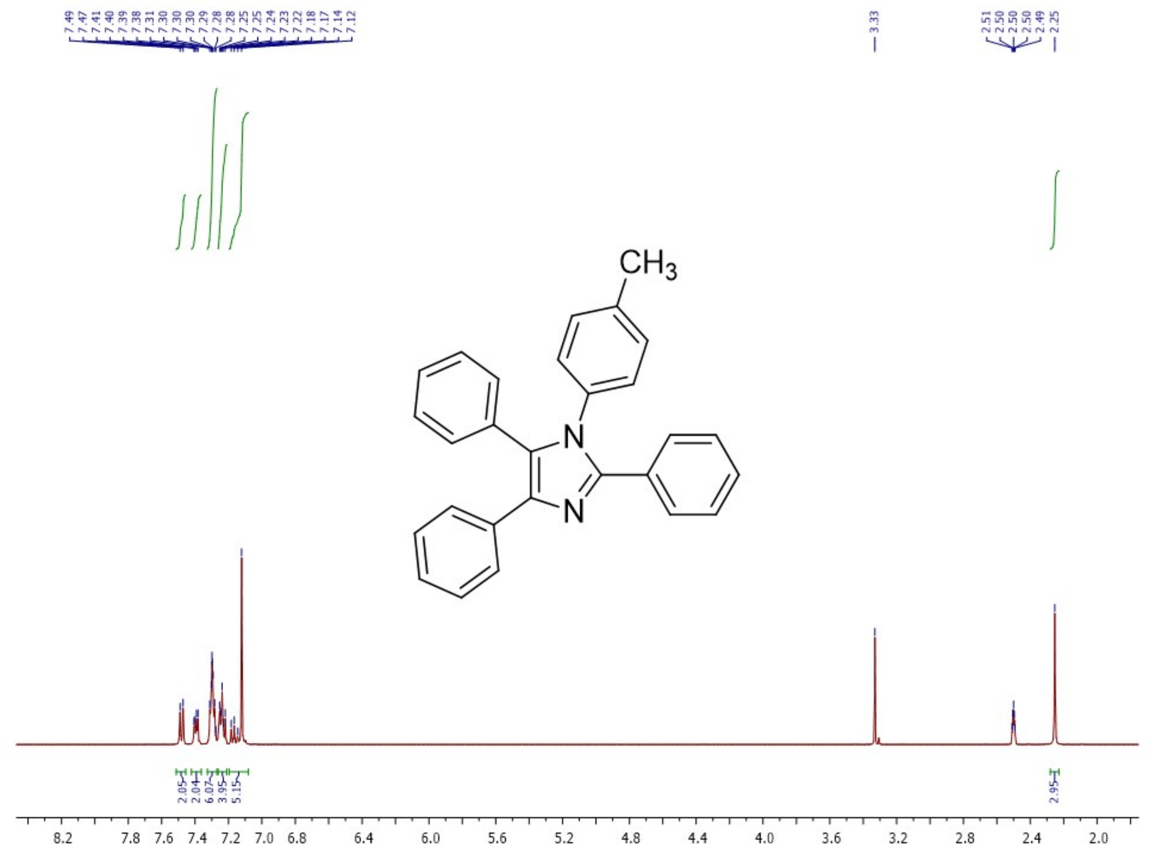


**$^{13}$C-NMR**

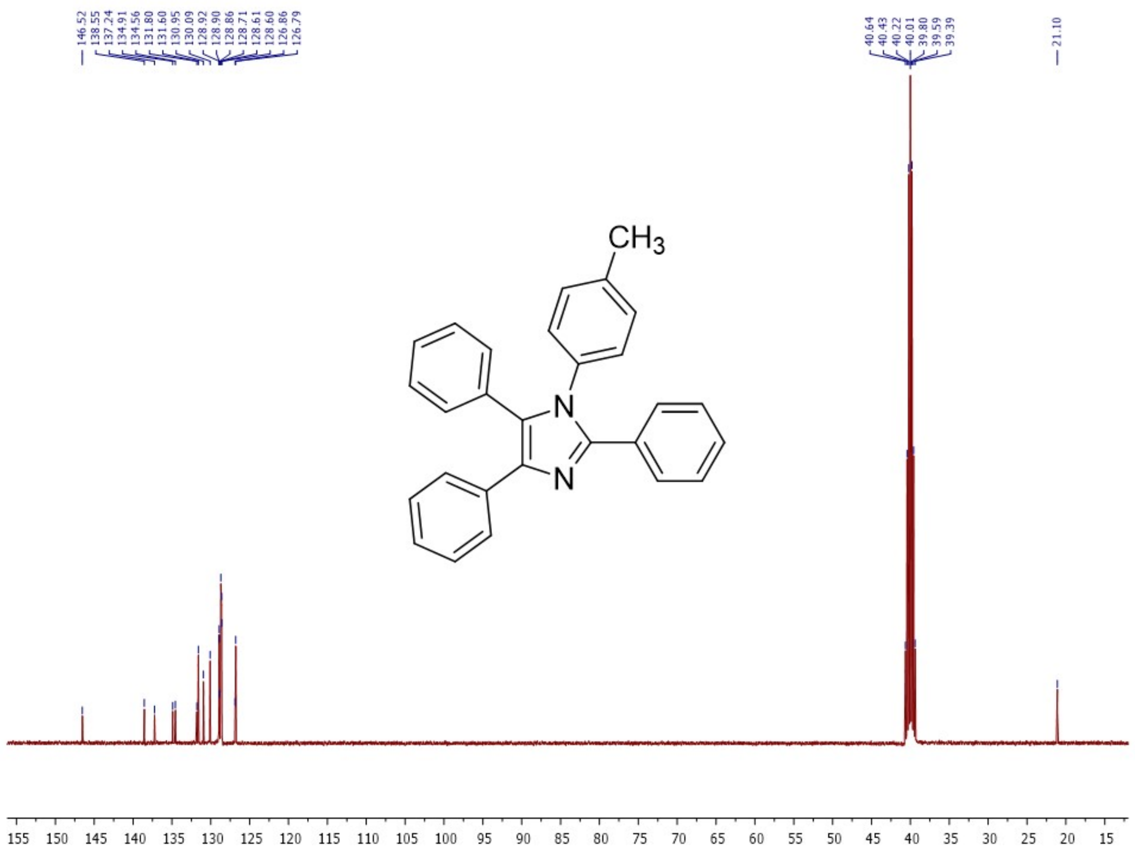


**Mass**

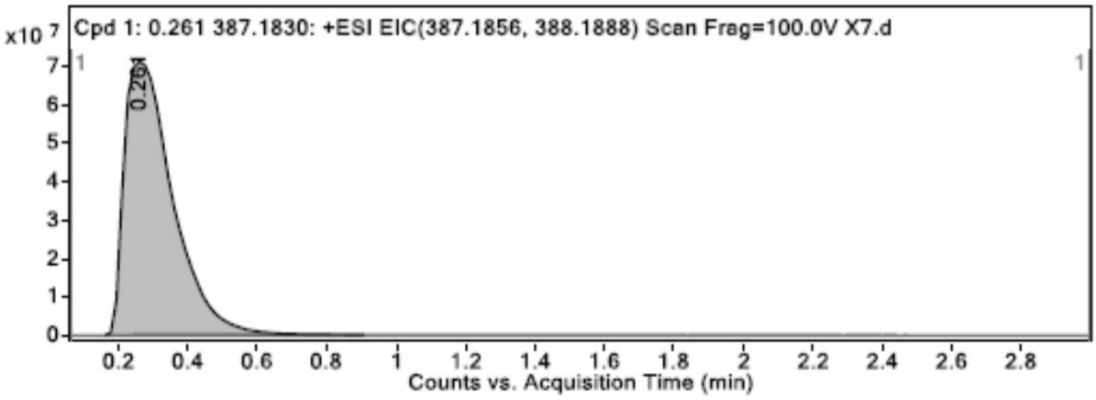


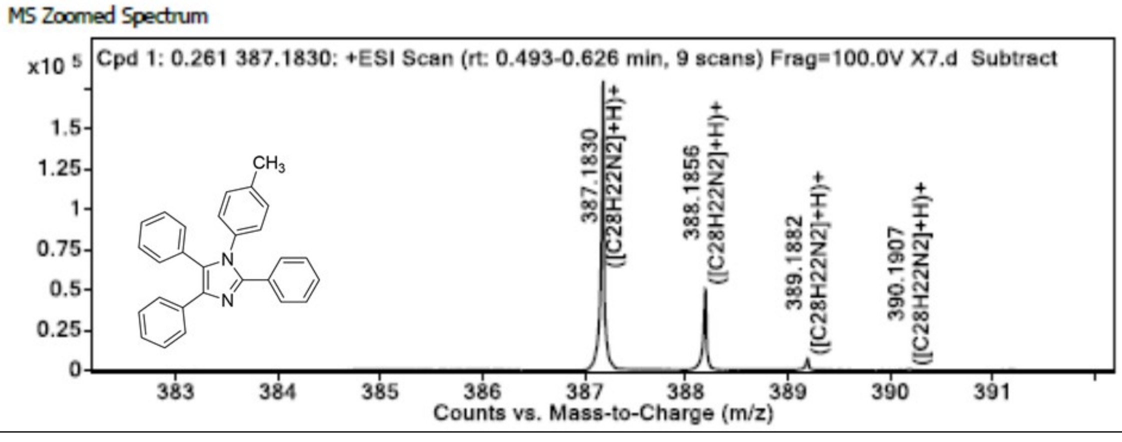


**2. Characterization of ZSM-11 zeolite catalyst.**

| |
|---|
| **XRD pattern of Synthesized ZSM-11 at various 18-48 hrs. times. (a) 18 hrs., (b) 24 hrs., (c) 36 hrs., & (d) 48 hrs.** |
| 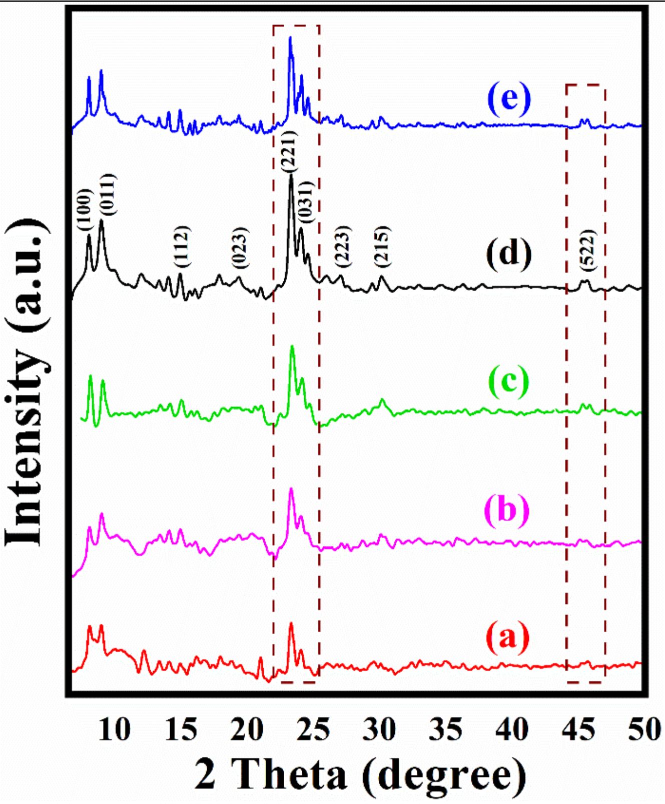  **Fig. 1** |
| **FTIR spectra of Synthesized ZSM-11 at various 18-48 hrs. times. (a) 18 hrs., (b) 24 hrs., (c) 36 hrs., & (d) 48 hrs.** |
| 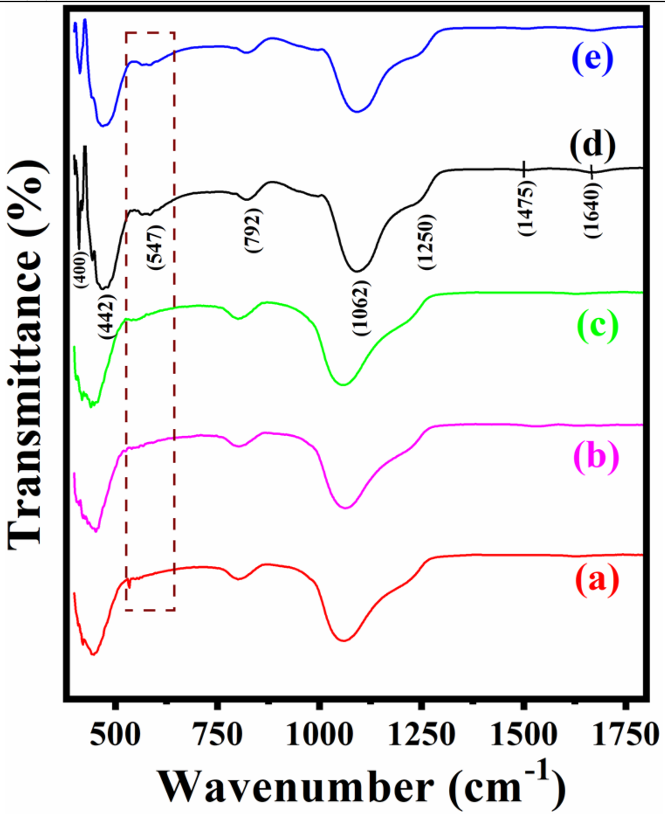  **Fig. 2** |

**FE-SEM images of synthesized at various 18-48 hrs. times (a) 18 hrs. (b) 24 hrs., (c) 36 hrs., & (d) 48 hrs.**

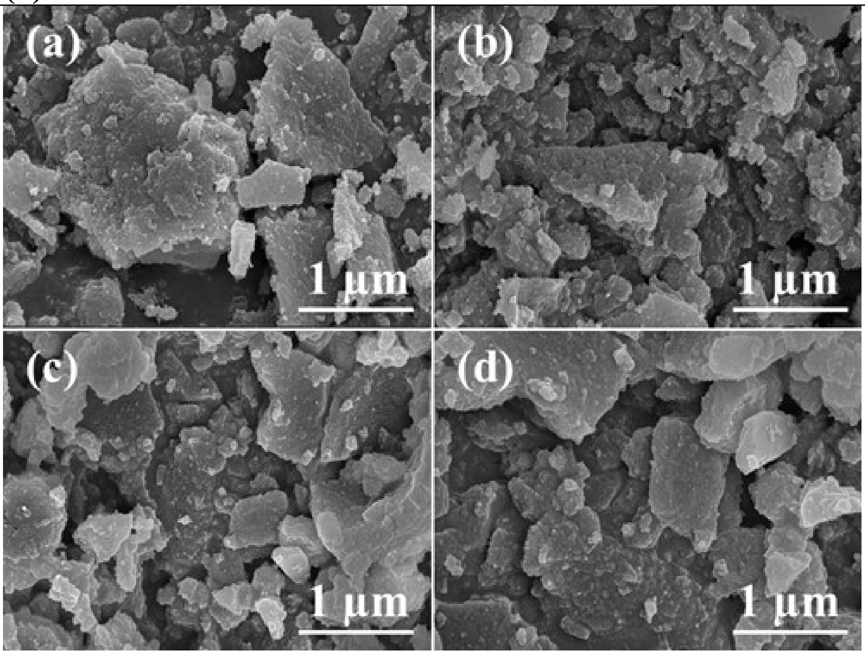


**Fig. 3**

**HR-TEM images of synthesized at various 18-48 hrs. times, (a) 18 hrs. (b) 24 hrs., (c) 36 hrs., & (d) 48 hrs.**

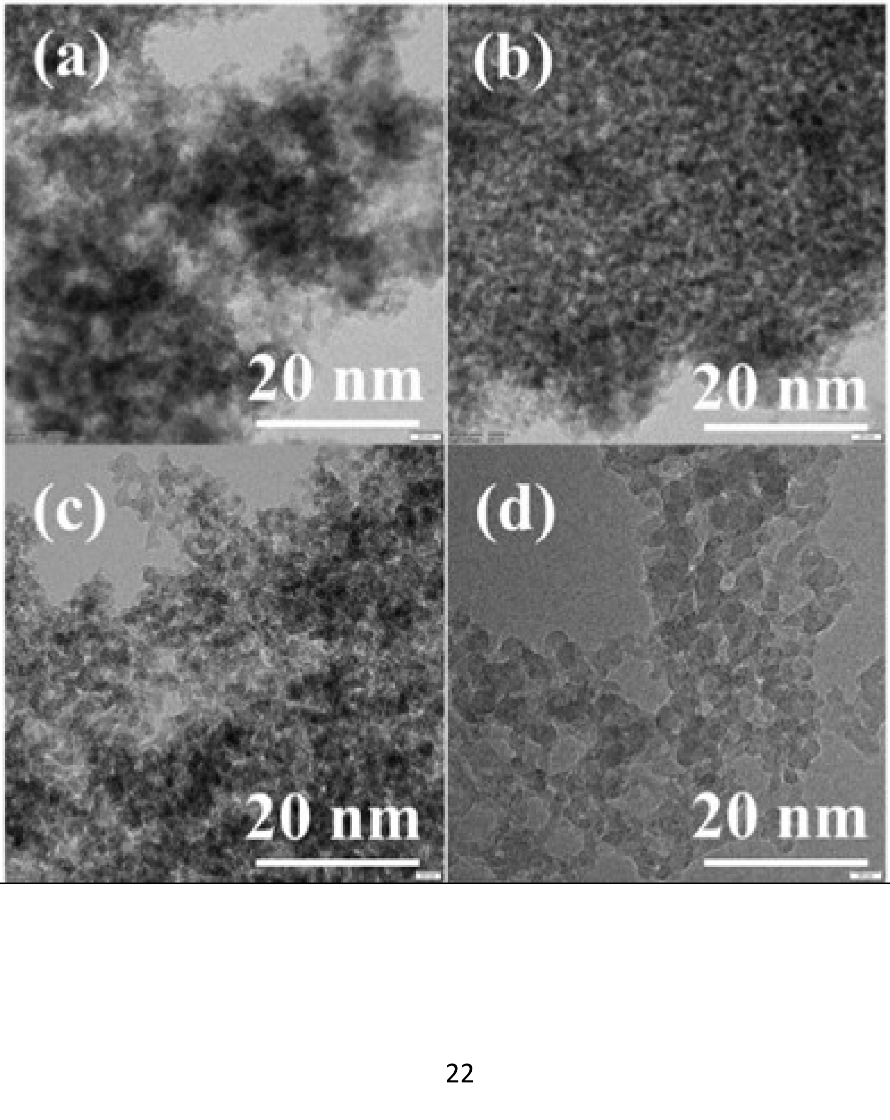

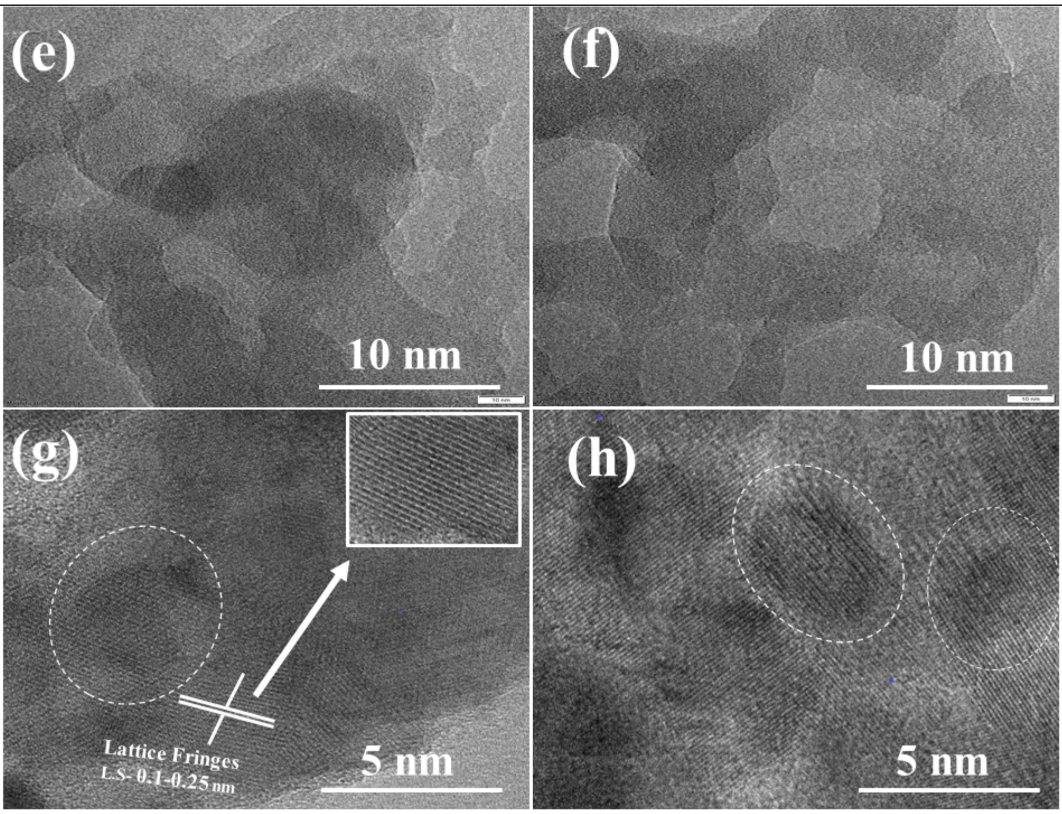


**Fig. 4**

**N2 adsorption-desorption isotherm of series of the ZSM-11 zeolite catalyst.**

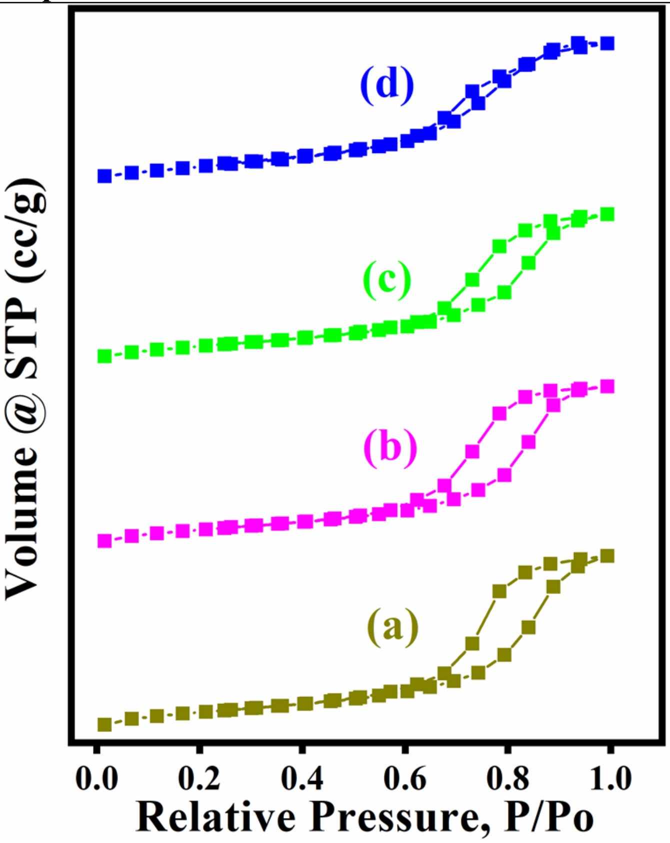


**Fig. 5**

**EDAX of series of ZSM -11 zeolite: ZSM-11-18 (a), ZSM-11-24 (b), ZSM-11-36 (c), and ZSM-11-48 (d). Elemental Mapping of ZSM-11 zeolite: ZSM-11-18 (e), ZSM-**

**11-24 (f), ZSM-11-36 (g), and ZSM-11-48 (h).**

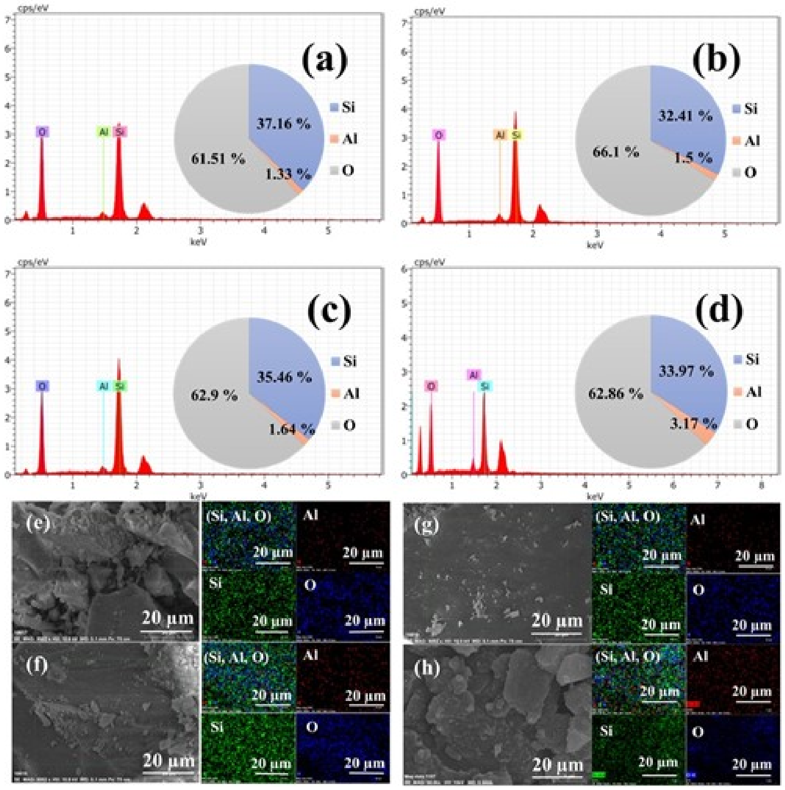


**Fig. 6**

**Pyridine IR spectroscopy of ZSM-11 zeolite samples. (a) 48 hrs., (b) 36 hrs., (c) 24 hrs., and (d) 18 hrs.**

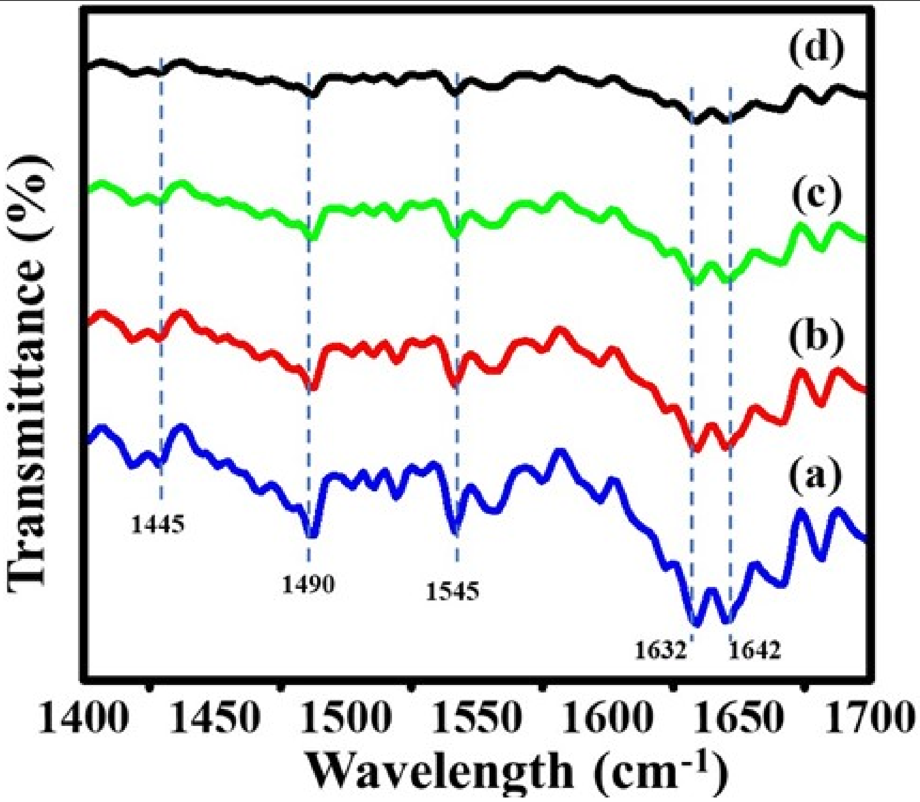


**Fig. 7**

**TGA/DTA Graph of ZSM-11 (48 Hrs.) zeolite**

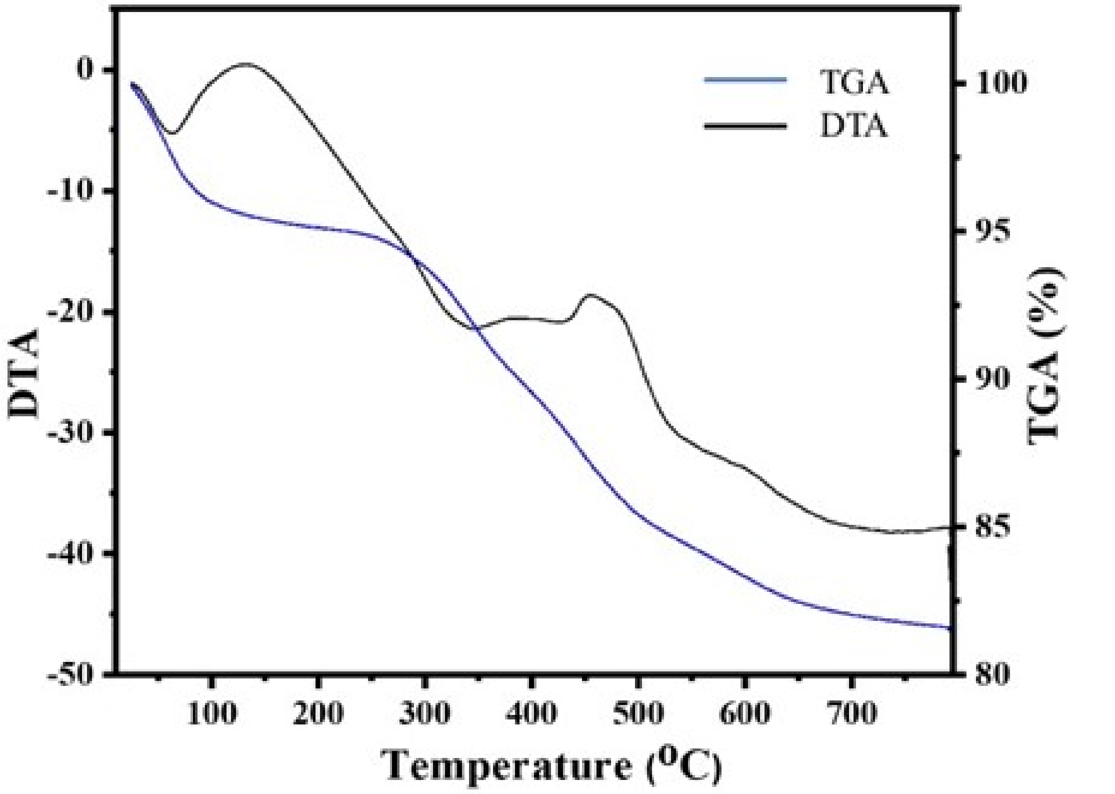
TGA
DTA
0
-10
-20
-30
-40
-50
DTA
100
95
90
85
80
TGA (%)
100
200
300
400
500
600
700
Temperature (°C)

**Fig. 8**
